# Response of Tomato Rhizosphere Bacteria to Root-Knot Nematodes, Fenamiphos and Sampling Time Shows Differential Effects on Low Level Taxa

**DOI:** 10.3389/fmicb.2020.00390

**Published:** 2020-03-20

**Authors:** Mariantonietta Colagiero, Laura Cristina Rosso, Domenico Catalano, Leonardo Schena, Aurelio Ciancio

**Affiliations:** ^1^Istituto per la Protezione Sostenibile delle Piante, Consiglio Nazionale delle Ricerche, Bari, Italy; ^2^Istituto di Tecnologie Biomediche, Consiglio Nazionale delle Ricerche, Bari, Italy; ^3^Department of Agriculture, Università degli Studi Mediterranea di Reggio Calabria, Reggio Calabria, Italy

**Keywords:** 16S rRNA, fenamiphos, *Meloidogyne*, metagenome, nematode, soil

## Abstract

A factorial taxonomic metabarcoding study was carried out to determine the effect of root-knot nematodes (*Meloidogyne incognita*, RKN) and the nematocide fenamiphos on the rhizosphere microbiome of tomato. Plants inoculated (or not) with RKN second-stage juveniles (J2), and treated (or not) with the nematocide, were tested in a 6 months greenhouse assay using a RKN-free soil proceeding from an organic crop. Rhizosphere soil was sampled at J2 inoculation, 3 months later (before the second nematocidal treatment), and again after 3 months. At each sampling, the RNAs were extracted and the 16S rRNA V4 regions sequenced with a Next Generation Sequencing (NGS) protocol. Changes in bacteria metagenomic profiles showed an effect of the treatments applied, with different representations of taxa in samples receiving nematodes and fenamiphos, at the two sampling times. In general, a tendence was observed toward an increase number of OTUs at 6 months, in all treatments. β-Proteobacteria were the most abundant class, for all treatments and times. When compared to soil before transplanting, the presence of tomato roots increased frequency of Actinobacteria and Thermoleophilia, reducing abundance of Solibacteres. At lowest taxonomic levels the samples clustered in groups congruent with the treatments applied, with OTUs differentially represented in relation to RKN and/or fenamiphos applications. *Bacillus*, *Corynebacterium, Streptococcus*, and *Staphylococcus* were more represented at 6 months in samples inoculated with RKN. The nematodes with the nematocide application increased the emergence of rare OTUs or reduced/enhanced the abundance of other taxa, from different lineages.

## Introduction

Bacteria underpin several specialized functions in soil food webs, including nutrient recycling, detoxification and regulation of pests, which may lead to macroscopic effects including pest or pathogens suppression (Esnard et al., 1995; Westphal and Becker, 2001; Westphal and Xing, 2011). Several species, moreover, have a biotechnological potential, including antibiosis (Schloss and Handelsman, 2003). The diversity and densities of the operational taxonomic units (OTUs) present in the rhizosphere depend, among other factors, also on the bacteria specialization and trophism. Moreover, factors such as the aboveground vegetation and soil physiochemical properties affect the composition and stability of belowground bacterial communities, as well as reflecting farming practices (Leff and Fierer, 2013). Diversity of soil bacteria is also influenced by local microhabitat conditions, which may vary over very small scales. Other significant factors, however, have direct anthropic origins, including a number of agricultural practices such as the addition of fertilizers and organic matter, the application of chemicals for pest and weed management and the accumulation of related by-products (Foster, 1988; Van Elsas et al., 2008; Souza et al., 2013). The telluric microbial diversity is in the order of thousand OTUs ⋅ g^–1^ of soil, of which only a small fraction (0.1–1%) is suitable for cultivation and isolation. This leaves the effects of several taxa mostly undetermined (Kellenberger, 2001; Rosello-Mora and Amann, 2001; Roesch et al., 2007).

This situation requires effective and sensitive techniques to identify the services provided by soil bacteria or to evaluate the impact of factors such as common agricultural practices and presence of pests. The study of diseased roots may yield informations on the shifts in bacterial community composition related to parasitism (Tian et al., 2015). Similarly, also the chemicals applied for disease or pest management may affect the rhizosphere bacterial diversity or induce changes in the physiology of roots, with indirect effects on specialized rhizosphere taxa such as growth promoters or biocontrol agents.

Nematodes are a key component of soil food webs, including pest species that have a direct impact on plants productivity and yields. For decades nematode pests have been controlled through the application of a wide range of chemicals, including fumigants, carbamates or organophosporous compounds. Due to many inconvenients related to the use of chemical nematocides, including soil, groundwater contamination and toxicity to higher animals including man, several chemicals have been banned or limited for use. Among organophosphorous products, fenamiphos has been used as a nematocide and insecticide. It has been widely applied against nematodes at concentrations varying around 10 kg of active ingredient (a.i.) ⋅ ha^–1^. Actually, in the EU it may be only applied in permanent greenhouses through drip irrigation, halting treatments at least 60 days before starting harvesting. Fenamiphos, that is effective systemically or by contact against most nematode pests through acetylcholinesterase inhibition (Ravichandra, 2018), may be degraded by a number of soil bacteria (Megaraj et al., 2003; Cáceres et al., 2008; Cabrera et al., 2010).

The relationships of nematodes with bacteria likely cover all type of interactions, ranging from symbiosis to antagonism. Although the studies on these interactions are, given the large rhizosphere bacterial diversity, still incomplete, a number of taxa have been reported in microbiome studies of nematodes (Ladygina et al., 2009; Tian et al., 2015; Elhady et al., 2017; Hussain et al., 2018; Hu et al., 2019). The diversity of clades associated to soil nematodes has been mostly explored using the 16S ribosomal rRNA gene sequence. Variations in the microbiome composition related to parasitism were shown by comparing nematode-parasitized and healthy tomato roots (Tian et al., 2015). Metagenomics of the pinewood nematode (PWN) *Bursaphlenchus xylophilus* showed that mutualistic symbiotic bacteria provided a detoxification function, allowing PWN to survive the host pine defense reaction (Cheng et al., 2013). Bacteria directly linked to nematodes include species found in association to free-living or plant parasitic species, such as *Pseudomonas* spp., Verrucomicrobia, or endosymbionts such as *Cardinium*, *Wolbachia*, or *Pasteuria* spp. (Noel and Atibalentja, 2006; Haegemann et al., 2009; Zou et al., 2015; Hussain et al., 2018). Members of Bacillales in association to nematodes include *Bacillus nematocida*, described from free-living *Panagrellus redivivus* (Huang et al., 2005), or species active against *Caenorhabditis elegans* and *Pristionchus pacificus* (Rae et al., 2010).

This study was motivated by the need to enlarge actual knowledge on the interactions among rhizosphere bacterial communities, nematode pests and nematocides applied for their management. Starting hypothesis was that either the nematode presence and the application of a nematocide affect the composition, reproduction and activity of rhizosphere bacterial communities. One possibility considered was a change in the abundance of unknown taxa, directly acting as nematode endosymbionts or antagonists, as well as related to plant parasitism, or indirectly linked to nematode metabolism. A second effect hypothesized was an effect on bacterial groups in relation to a nematocide treatment or an increase of taxa involved in its decomposition. Finally, it is not known whether and at which extent (i.e., acting on single species or on entire clades) these effects could be observed, as well as how long they persist in time.

In particular, we aimed at identifying the effect of the root-knot nematode (RKN) *Meloidogyne incognita* and/or fenamiphos on the diversity of bacteria in tomato rhizosphere. We examined the changes induced on the bacterial community composition in the rhizosphere of plants growing in a previously untreated and pest-free soil, proceeding from an organic farm. Being culture-independent, taxonomic metabarcoding techniques allow a broad identification range and a sufficient sampling depth, representing the standard for analysis of microbial communities in many environments (Handelsman, 2004; Patrick and Handelsman, 2005; Susannah and Edward, 2005; Caporaso et al., 2010). A taxonomic metabarcoding NGS-based approach was hence applied to study the changes in bacterial diversity in treated plants, in a long lasting (more than 6 months) greenhouse experiment, by measuring rhizosphere OTUs abundance, at two different sampling times.

## Materials and Methods

Soil (53.9% sand, 14.4% silt, 31.7% clay, pH 7.8) was collected in July 2012 at 20–30 cm depth from a single field sampling site, ca. 1 m wide, from a parcel not cultivated for 2 years in an organic horticultural farm located at Mesagne (Brindisi, Italy), and only superficially labored to remove weeds. Soil depth was chosen to get soil as uniform as possible, avoiding parts exposed to sunlight or dry, presence of debris, and irregularities in the soil surface profile. The samples were checked for presence/absence of RKNs by the soil sieving and decanting technique, suspending a 2 l soil sub-sample in tap water and filtering with a set of 500 and 75 μm sieves. The filtered suspension was then examined in a Hawksley counting chamber with a Leitz Orthoplan light microscope at 50×, in three replicates. After no RKN nematode stage was detected (only free living nematodes were found), the remaining soil was mixed, distributed in 20 cm diam. pots, kept in a greenhouse and all planted with seedlings of *Solanum lycopersicum* L. cv Tondino previously germinated in turf and washed free of the substrate residues.

The assay consisted of four treatments sampled at two intervals of 3 months each (Supplementary Figure S1A). The treatments were: (i) soil untreated and free of RKN (control); (ii) the same soil inoculated with RKN juveniles (J2); (iii) the same soil inoculated with the J2 and treated with the nematocide fenamiphos and (iv) the same soil treated only with fenamiphos. The nematocide commercial product used (Nemacur^®^ 240 CS, with 23.1% a.i.) is a micro-encapsulated formulation applied for vegetable crops such as tomato at 42 l ⋅ ha^–1^. At each treatment, 0.13 ml of product were applied per pot, calculated for the pot surface (10 cm radius) using the 4.2 ml ⋅ m^–2^ dose indicated by the producer, corresponding to 0.03 ml of a.i. per pot.

The J2 used (*M. incognita* population L4, descending from a single egg mass and proceeding from Leverano, Lecce) were multiplied on tomato plants cv Tondino in artificially infested soil, originally sterile. To obtain the J2, fragmented root galls were placed in flasks with sterile water (SW) and aerated with a peristaltic pump to allow eggs hatching. The freshly hatched J2 were washed and superficially sterilized in 0.5% NaOH hypochloride for 5 min, followed by washing in an antibiotic solution (0.1% streptomycin, 0.1% ampicillin, 0.1% chloramphenicol) for 10 min, and then in SW for further 10 min. Before seedlings inoculation, the soil of two control pots was stirred before collecting the first (time 0 = T_0_) 2 g samples, that were then frozen in liquid nitrogen and stored at −80°C, for subsequent RNA extraction. The nematode inoculations were then carried out by adding 5000 J2 per pot through gentle pipetting. The J2 number was adjusted in 25 ml of sterile water suspension containing 200 J2⋅ ml^–1^, that were pipetted around the plant base in three equally spaced points, at a depth of about 8–10 cm. Each treatment was planned in five replicated pots, using three of them for the taxonomic metabarcoding analyses. The test was carried out in a greenhouse under controlled conditions (26 ± 2°C, RH 40–60%), with two further sampling times, T_1_ = 90 days post inoculation (dpi) and T_2_ = 180 dpi, after the initial baseline T_0_ (Supplementary Figure S1A).

One month after inoculation, the plants were checked for galls to confirm RKN infestation. At this point, the seedlings of treatments including the nematocide application were treated twice (at end of month 1 and 2) with fenamiphos, as described. One month later, the second series of rhizosphere soil samples (T_1_) was collected from roots. The roots were excavated and exposed in each pot using a sterile steel spatula, collecting 2–3 g of soil particles and aggregates at a distance within 5 mm from the exposed root and/or gall surface, avoiding any emerging tip (for sampling timing see experimental scheme in Supplementary Figure S1A). Data on gall index, plant length, root weight, density of eggs ⋅ g^–1^ of roots were collected. The assay continued by transplanting in each pot new tomato seedlings at the third leaf stage, collecting the third and final rhizosphere soil samples after three further months (T_2_).

Total RNA was extracted from three replicated rhizosphere soil samples (each from a single pot), per treatment and time, with the PowerSoil Kit (MoBio Laboratories, CA), following the manufacturer’s instructions. The RNA concentration was determined with a Nanodrop^TM^ spectrometer at 260 nm. The extracted material was subjected to reverse transcription according to the Illumina^TM^ sequencing protocol, using SuperScript III (Invitrogen, United States), following the manufacture’s protocol. The material obtained was then purified using the QIAquick PCR Purification Kit (Qiagen^®^, United Kingdom).

The nucleic acids integrity was checked by electrophoresis on 1.5% agarose gel. The subsequent taxonomic metabarcoding analyses relied on the bacterial 16S ribosomal rRNA (rRNA) gene sequence data, produced from the 16S hypervariable regions that may be amplified using universal primers with affinity for flanking conserved motifs (Van de Peer et al., 1996; Baker et al., 2003; Clarridge, 2004). In the 16S rRNA nine “hypervariable regions” (V1–V9) are present that show enough diversity to discriminate at the species or genera levels (Kataoka et al., 1997; Bertilsson et al., 2002; Becker et al., 2004).

MiSeq System^TM^ Illumina platforms, provided by commercial services (IGA-Technology, Udine and Genomix4life, Salerno, Italy), were used for sequencing the samples, in separate runs. Both ends of the V4 16S rRNA hypervariable region were used (Yang et al., 2002). They are considered capable to yield sufficient informations for taxonomic classification (Liu et al., 2007, 2008; Caporaso et al., 2011). In all bacteria the V4 region consists of 254 nt differing only for a few base pairs. According to the Illumina protocol this region was amplified with 515F (5′ GTGCCAGCMGCCGCGGTAA 3′) and 806R (5′ GGACTACVSGGGTATCTAAT 3′) primers, with anchored adapters unique for each sample, for subsequent identification.

For the bioinformatic assembly of the single read contigs, PandaSeq^1^ was used (Masella et al., 2012), with the following parameters/conditions: filtering sequences with unidentified nucleotides; minimal length of overlapping region = 25 nt; contig lengths (min-max) = 245–265 nt (Claesson et al., 2010). For each sample, the single fasta format file of assembled sequences obtained, by merging data, was then used as first input for data processing with QIIME, running in a Linux emulator (Virtual box), within a Windows 7 environment.

QIIME was applied to analyze the OTUs assigned through the implementation of UCLUST, applying a 97% identity threshold to discriminate at the species level (Caporaso et al., 2010). An OTU table was then constructed using *pick_de_novo_otus.py* and a combined fasta file with labels, generated by a metadata mapping file with *add_qiime_labels.py*. A biom-formatted OTU-table was then obtained, and used for analyses. Selected representative sequences of each OTU were classified using a QIIME-based wrapper of the Ribosomal Database Project (RDP) classifier, using a 0.80 confidence threshold for taxonomic assignments and the RDP core set (Wang et al., 2007; Cole et al., 2009; Edgar, 2010). β-diversity estimates were calculated with QIIME using weighted Unifrac distances between samples (Lozupone and Knight, 2005), at a depth of 6000 sequences per sample. Jackknifed principal coordinates (PCoA) were then computed. PAST (Hammer et al., 2001) was used to calculate a set of OTU α-diversity indices.

The OTU biom format file with sequence abundance per sample and treatments was analyzed with the graphical interface provided by STAMP (Statistical Analysis of Metagenomic Profiles ver. 2.1.3)^2^ (Parks and Beiko, 2010; Parks et al., 2014). To compare sample pairs or samples organized into two or more groups identified by treatment and/or other traits provided with the mapping file (such as sampling time, presence/absence of nematodes or nematocide), the entire samples were used as parent level with different profile levels, applying a two tailed Student’s *t*-test, with other comparative statistics. To keep unclassified OTUs and their higher levels in the analyses, the latter were identified in the hierarchy (and eventually represented in plots) by using the corresponding OTUs ID as tags for the higher taxonomic levels. Heatmap plots of only active features (ANOVA, with 0.95 *post hoc* Tuckey–Kramer test, filtering threshold: *P* ≤ 0.05) were produced with the average neighbor UPGMA algorithm and a 0.65 dendrogram clustering threshold. Two groups comparisons were performed applying a two sided, equal variance *t*-test (*P* ≤ 0.05, effect size as ratio of proportions = 0.8). Metagenome ring-charts by treatments and sampling time were produced with Krona (Ondov et al., 2011). Further analyses were performed using R (R Core Team, 2013), with Bioconductor libraries *Biobase*, *BiocParallel*, *BiocVersion* (Ihaka and Gentleman, 1996), *ggplot2* (Wickham, 2016) and package *mctoolsr*, ver. 0.1.1.2^3^.

## Results

### Bacterial Microbiome Composition and Diversity

A total of 13.5 ⋅ 10^6^ single reads were obtained from the 25 samples analyzed, yielding 6.6 ⋅ 10^6^ qualitatively valid sequences with an average length of 93 bp and 56% GC content (for raw data inventory see NCBI Bioproject PRJNA371772)^4^. PandaSeq yielded 483687 contigs that were analyzed with QIIME, yielding a total 294597 sequences, that were used to get the taxonomic assignments in each sample.

**FIGURE 1:**
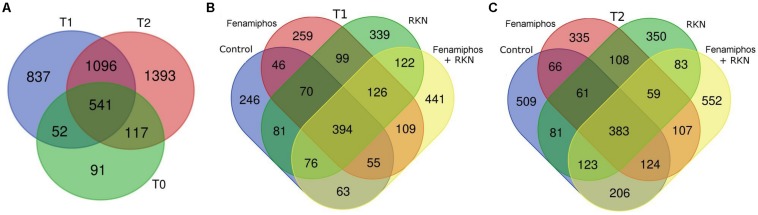
Venn diagrams showing the number of OTUs in all samples before planting (T_0_) and at 3 (T_1_) and 6 months (T_2_) **(A)**. Number of unique and shared OTUs by treatment, 3 (T_1_, **B**) and 6 months (T_2_, **C**) after pre-transplant sampling.

The 4127 OTUs obtained (including 79 redundants) were represented in the various samples with different frequencies. Only 93 (2.3%) of them were classified at the species level. The OTUs belonged to 179 families, 125 orders, 79 classes and 28 bacterial phyla, with Archea only represented by phylum Crenarchaeota (Supplementary Figure S2). A trend toward an increased number of total and unique genera was observed at T_2_ in all treatments, with fenamiphos plus RKN showing the highest score (Supplementary Figures S1B, S3). Similarly, also the total number of OTUs progressively increased during the 6 months of the experiment, as did the number of unique OTUs, that increased from T_1_ to T_2_, in all treatments (Figures 1A–C).

**FIGURE 2:**
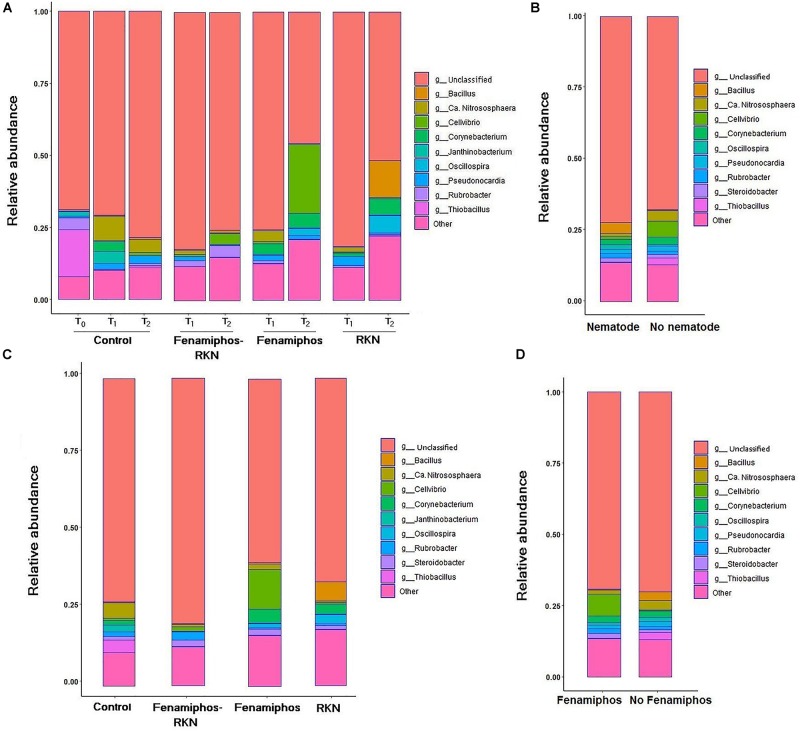
Relative abundance of the ten most represented genera, by treatment and sampling time **(A)**, when considering pooled samples with or without *Meloidogyne incognita* (RKN) inoculum **(B)**, pooled by treatment **(C)**, and with or without fenamiphos application **(D)**.

β-Proteobacteria were the most abundant class, for all treatments and times. The most represented lineages were α-, β-, γ-, and δ-Proteobacteria, which accounted for 66% of sequences at T_0_, followed by Actinobacteria, Acidobacteria, Gemmatimonadetes and others. When compared to soil before transplanting, the presence of tomato roots increased frequency of Actinobacteria and Thermoleophilia, reducing abundance of Solibacteres (Table 1 and Supplementary Figure S2). After rarefaction at 800 sequences, which kept all samples, the first ten taxa with highest relative abundance (mean% of total) showed different frequencies per treatment and time, with a number of dominant genera. Most represented in the control samples were genera *Thiobacillus* (at sampling time T_0_), and the archean *Ca.* “Nitrososphaera” (at T_1_ and T_2_). *Cellvibrio* showed a dramatic increase in time in treatments with fenamiphos, whereas *Bacillus* was dominant at T_2_ in the samples inoculated with RKN only, followed by *Oscillospira* and *Corynebacterium* (Figure 2A). When considering presence/absence of RKN, *Bacillus* again was dominant in presence of RKN, whereas *Ca.* “Nitrososphaera” and *Cellvibrio* were most abundant in samples without RKN inoculation, followed by *Corynebacterium* and *Thiobacillus* (Figure 2B).

**TABLE 1:** Relative abundance (% of total) of sequences for most common bacterial phyla and classes, by treatment and time.

**Phylum**	**Class**	Treatments*									**Mean**
			
		**Control**			**RKN**		**Fenamiphos**		**Fenamiphos and RKN**		
					
		**T_0_**	**T_1_**	**T_2_**	**T_1_**	**T_2_**	**T_1_**	**T_2_**	**T_1_**	**T_2_**	
**Proteobacteria**											
	α-Proteobacteria	6	4	6	5	3	5	5	11	6	5.67
	β-Proteobacteria	51	31	13	11	19	16	11	10	37	22.11
	γ-Proteobacteria	3	5	6	7	7	9	48	6	9	11.11
	δ-Proteobacteria	6	2	5	2	3	6	3	7	6	4.44
**Actinobacteria**											
	Actinobacteria	4	16	12	12	12	13	10	11	8	10.89
	Acidimicrobiia	2	2	4	4	5	3	4	3	8	3.89
	Thermoleophilia	2	18	13	10	5	6	2	5	4	7.22
	Rubrobacteria	5	0.5	1	0.7	0.4	0.8	0.3	2	2	1.41
**Gemmatimonadetes**											
	Gemm-2	0.1	0.4	0.8	0.4	1	0.2	0.2	1	0.9	0.56
	Gemm-3	0.3	0.4	0.6	0.6	0.9	0.3	0.3	0.6	0.4	0.49
	Gemm-5	0.2	1	2	2	3	0.9	1	0.6	1	1.30
	Gemmatimonadetes	1	2	7	3	5	3	2	0.9	5	3.21
**Armatimonadetes**											
	Fimbriimonadia	0.1	0.04	0.09	0.1	0.06	0.04	3	0.7	0.1	0.47
	c_0319-6E2	0.05	0.06	0.3	0.7	–	0.6	–	1	0.01	0.39
**Acidobacteria**											
	Acidobacteriia	0.07	0.2	0.2	1	0.1	6	0.03	2	0.1	1.08
	Solibacteres	15	3	10	13	6	7	2	10	6	8.00
	[Chloracidobacteria]	0.2	1	5	8	0.08	7	0.2	10	0.09	3.51
**Chloroflexi**											
	Chloroflexi	0.03	0.3	0.9	0.8	0.02	0.6	0.007	0.9	0.01	0.40
	Ellin6529	0.03	0.1	0.3	0.5	0.03	0.5	0.03	0.8	0.005	0.26
	Thermomicrobia	0.08	0.6	1	2	0.1	2	0.07	3	0.09	0.99
**Nitrospirae**											
	Nitrospira	1	1	2	7	3	5	0.7	4	3	2.97
**Firmicutes**											
	Clostridia	–	0.04	0.06	0.1	5	0.01	3	0.04	0.02	1.03
	Bacilli	1	0.7	1	1	16	1	1	1	1	2.63

*Each treatment mean of three replicates, except Control at T_0_ and Fenamiphos with RKN at T_2_, with two replicates each.

Sequence data pooled by treatment showed *Ca.* “Nitrososphaera” and *Thiobacillus* as most prevalent in control, *Cellvibrio* and *Corynebacterium* in fenamiphos, *Rubrobacter* in fenamiphos with RKN, and *Bacillus* with *Corynebacterium* and *Oscillospira* in samples with only RKN (Figure 2C). Comparing samples by presence/absence of the nematocide showed *Cellvibrio* and *Steroidobacter* as more represented in presence of Fenamiphos, whereas *Bacillus*, *Ca.* “Nitrososphaera” and *Thiobacillus* were more represented in the other samples (Figure 2D).

**FIGURE 3:**
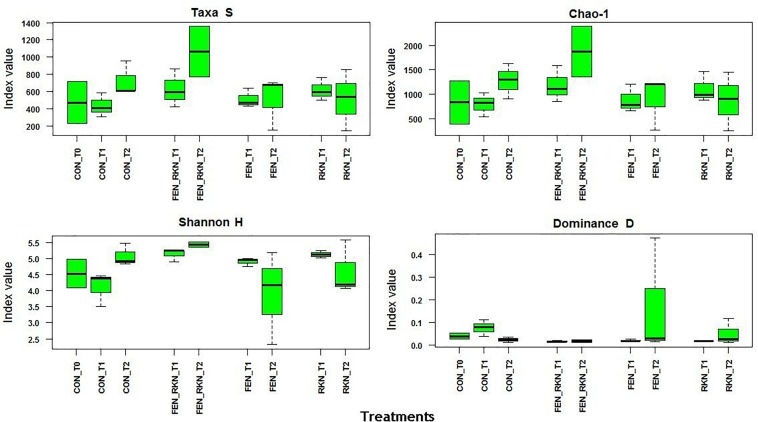
Alpha diversity indices selected for treatment and sampling times. Treatments codes: CON, untreated control; RKN, inoculated with *Meloidogyne incognita*; FEN, treated with fenamiphos; FEN-RKN, inoculated with *M. incognita* and fenamiphos. Boxes show the quartile range (75th to 25th). The median is shown as a line within the box. Whiskers indicate the most extreme data point within 1.5 ⋅ (75th – 25th percentile) of the median.

Mean α-diversity indices, calculated by PAST for treatments and times, showed higher values at T_2_ for the treatments with fenamiphos plus RKN and RKN only, either in terms of number of taxa or Chao-1, the latter indicating higher prevalence of rare OTUs. This was also reflected by a higher evenness variability, as indicated by Shannon H and dominance, with all taxa more equally present in the other treatments (Figure 3). The extent of the β-diversity among all samples showed distinct clusters, formed by individual samples rather than treatments groupings, mostly visible on the PCoA plan formed by the first two axes (accounting for 56% of variance), with a higher aggregation for the RKN-inoculated samples on the other two factorial plans (Supplementary Figures S1C–E).

### Effects of Treatments on Plants

In the conditions of the assay, the fenamiphos applications did not significantly affect the tomato plants performance, apart from an increase observed for the root weight at 3 months (*P* < 0.05). In the treated and RKN-inoculated plants, the number of RKN eggs and root gall index (RGI) decreased at T_2_, with an opposite trend in the plants inoculated only with nematodes. Application of the nematocide increased plants height at T_1_ (fenamiphos only), and at T_2_ in the treated samples, with nematode inoculation (Supplementary Figure S4).

### Bacterial Microbiome Evolution in Time

The abundance of taxa showed a number of changes in relation to time and treatments, when compared with the proportions in the pre-treatment control (T_0_) (Supplementary Table S1). Archea (Crenarchaeota), that were not detected at T_0_, were found at 3 months in control (8% of total sequences), in one sample treated with fenamiphos and, with lower proportions (0 – 1%), in other samples. They persisted at 6 months (T_2_) only in the untreated control, although with a mean reduction to 4% of total sequences. When Archea were present, *Candidatus* “Nitrososphaera” always accounted for more than 80% of their sequences (for Krona diagrams with mean proportions of taxa by treatments and times see Supplementary Figure S2).

**FIGURE 4:**
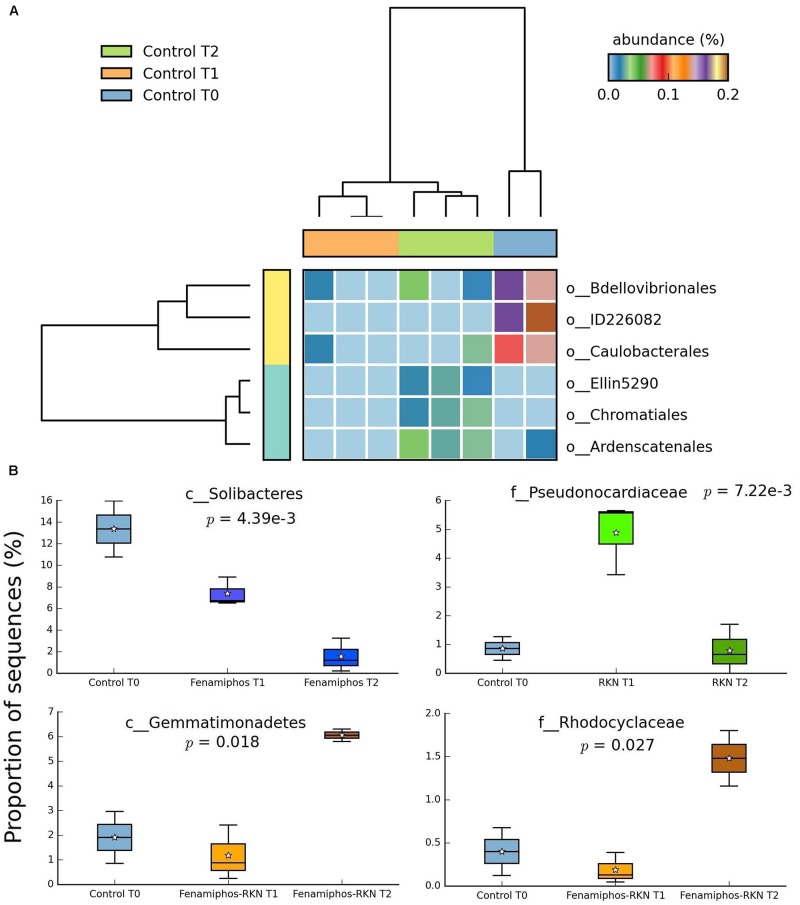
Significant changes (*P* < 0.05; effect size, ratio of proportions = 0.8) observed at the three sampling times for control soil samples, at the order level **(A)**, and changes in proportion of sequences at class and family levels **(B)**. For whisker plot details see Figure 3 legend.

**FIGURE 5:**
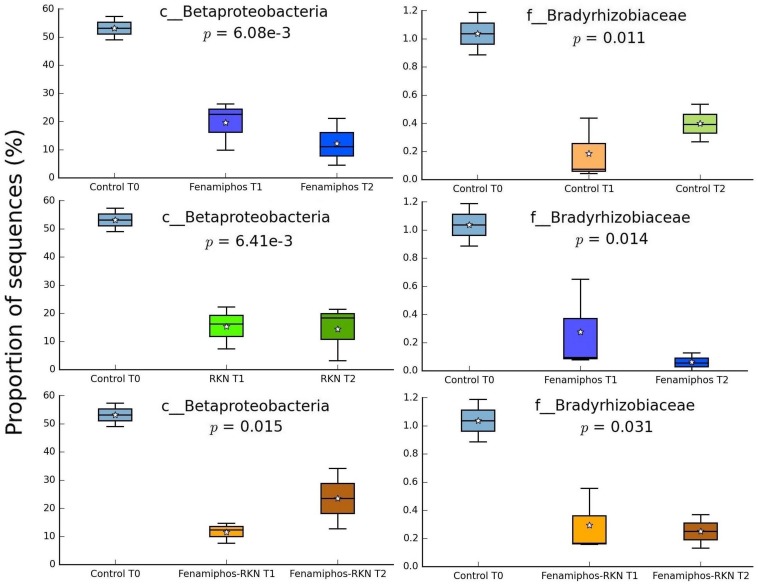
Changes of proportion of sequences (% of total) for β-Proteobacteria and Bradyrhizobiaceae, by treatment and sampling times. Boxes show the quartile range (75th to 25th). For whisker plot details see Figure 3 legend.

No significant change (statistical test: ANOVA; applied filtering criteria: *P* ≤ 0.05, effect size = 0.8, min. 1% of sequences at least in one sampling time) was found for the relative abundance of Bacteria in the control samples along the three sampling times, both at the phylum and class levels. At the order level, only six taxa forming congruent clusters were found along time accounting, however, for <0.3% of total sequences (Figure 4A). Significant differences were observed in time for β-Proteobacteria and Bradyrhizobiaceae (α-Proteobacteria) which, differing from the control, declined with fenamiphos, showing a more resilient response and an increase at T_2_, when RKN were also present (Figure 5).

**FIGURE 6:**
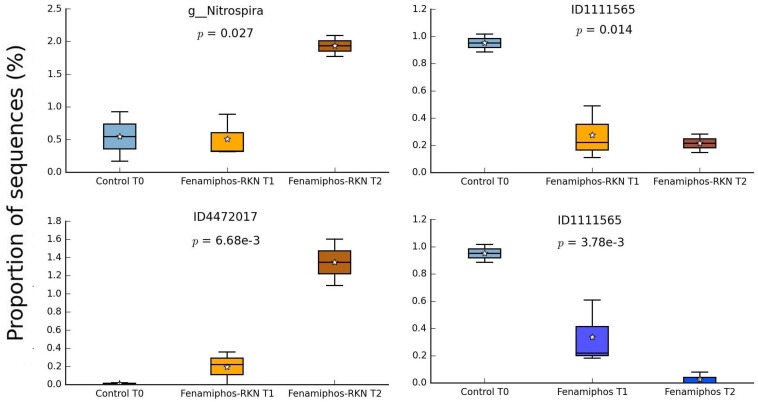
Changes in time of proportion of sequences (% of total; *P* ≤ 0.05; effect size, ratio of proportions = 0.8) for fenamiphos with RKN treatment, and comparison of OTU ID1111565 with trend observed in presence of fenamiphos only. Boxes show the quartile range (75th to 25th). For whisker plot details see Figure 3 legend.

**FIGURE 7:**
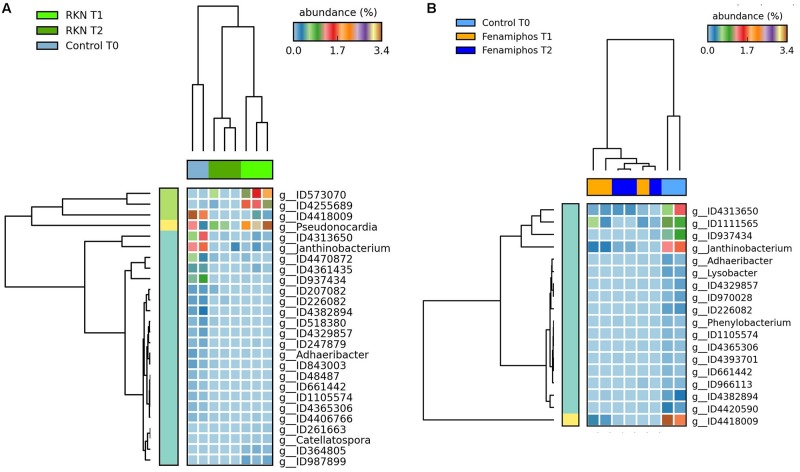
Heatmaps showing significant changes (% of total; *P* ≤ 0.05; effect size, ratio of proportions = 0.8) in abundance for genera significantly different at T_1_ and T_2_ from initial control (T_0_), for RKN-inoculated **(A)** and fenamiphos-treated samples **(B)**.

During the 6 months of the assay, RKN treatment showed an alternated trend for Pseudonocardiaceae (Actinobacteria) and, in combination with fenamiphos, increased Gemmatimonadetes and Rhodocyclaceae (β-Proteobacteria) (Figure 4B). Differing from control, the nematocide also had a detrimental effect on Solibacteres (Figure 4B), with OTU ID1111565 characterized by a more resilient response, when RKN were also inoculated (Figure 6). An opposite trend in time was shown by *Nitrospira* and OTU ID4472017 which, differing from control where their changes in time were not significant, sharply increased in presence of fenamiphos with RKN accounting for 1.8% of total sequences at T_2_ (Figure 6). When considering all samples together, number of unique OTUs was found mostly in this treatment, although with a low relative abundance (<1% of total sequences) (Supplementary Table S2). At the genus level the samples showed clusters distinct from control and, in the case of RKN inoculation, congruent with treatments and sampling times (Figures 7A,B).

### Effect of Treatments

**FIGURE 8:**
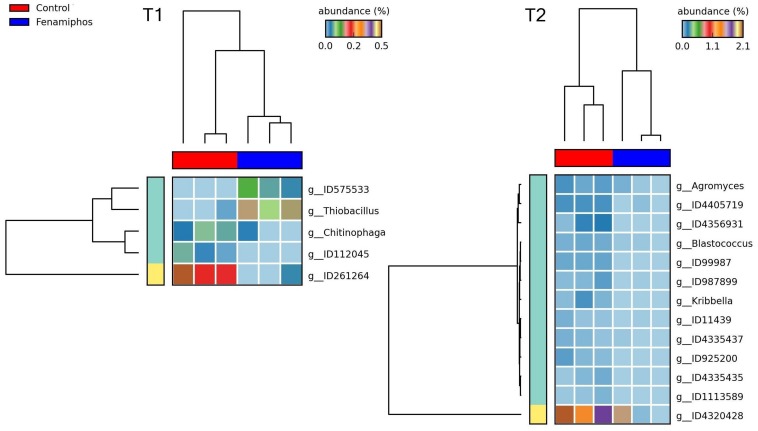
Heatmaps showing changes in abundance for genera significantly different, when comparing fenamiphos treatment with control, at T_1_ and T_2_.

**FIGURE 9:**
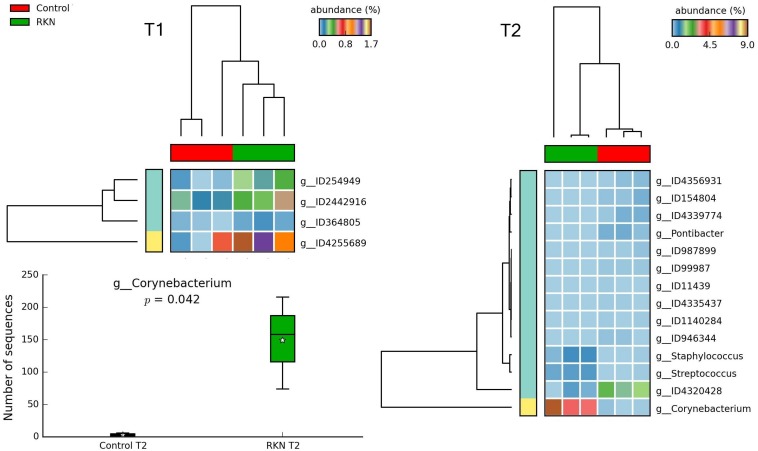
Heatmaps showing clustering of samples by treatment, with significant changes for abundance of genera (% of total; *P* ≤ 0.05; effect size, ratio of proportions = 0.8), when comparing RKN inoculation with control, at T_1_ and T_2_. Box plot shows differential abundance in the number of *Corynebacterium* sequences, at T_2_. For whisker plot details see Figure 3 legend.

**FIGURE 10:**
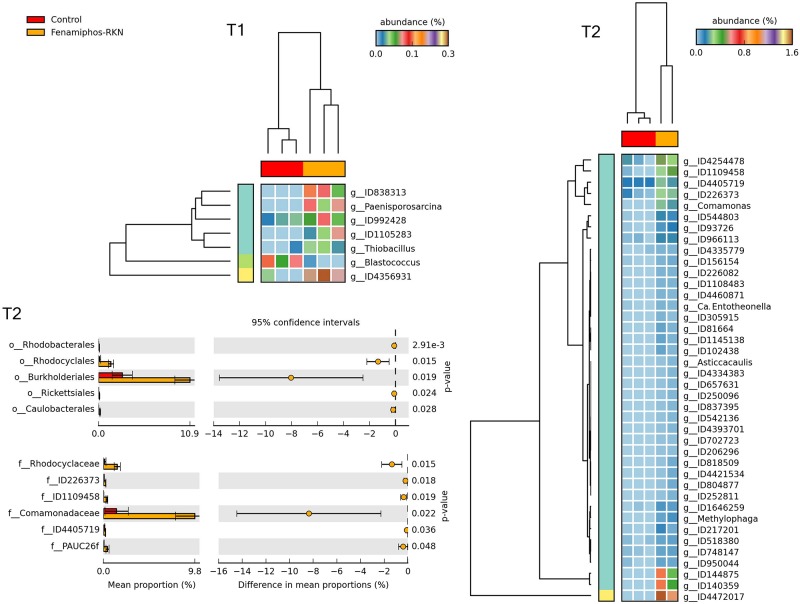
Heatmaps showing samples clustering by treatment with significant changes for abundance of genera (% of total; *P* ≤ 0.05; effect size, ratio of proportions = 0.8), when comparing samples of control vs. fenamiphos with RKN inoculation, and most significant changes in mean proportion (%) of sequences at the order and family levels, at T_2_ (equal variance *t*-test, two sided; bars show SD).

STAMP analysis showed changes in the relative abundance of taxa, reflecting the treatments and the time of sampling. Heatmaps showed samples that often clustered in groups congruent with treatments. In general, more taxa were identified at T_2_ as differentially represented among treatments, mostly at the genus level. Comparison with control of the fenamiphos-treated samples (two sided *t*-test equal variance, *P* ≤ 0.05, sequence filter maximum = 3–10; effect size filter as ratio of proportions = 0.8) showed differential abundance only at the genus level, at both sampling times, but for different taxa (Figure 8). Compared to control, the RKN-inoculated samples showed at T_2_ a higher relative abundance for *Corynebacterium*, *Streptococcus* and *Staphylococcus*, together with additional OTUs (Figure 9). A higher abundance of taxa was also observed at T_2_ in the samples treated with fenamiphos and inoculated with nematodes, when compared to control, involving mostly Proteobacteria, with a higher representation for Burkholderiales, with family Comamonadaceae (Figure 10 and Supplementary Table S3).

**FIGURE 11:**
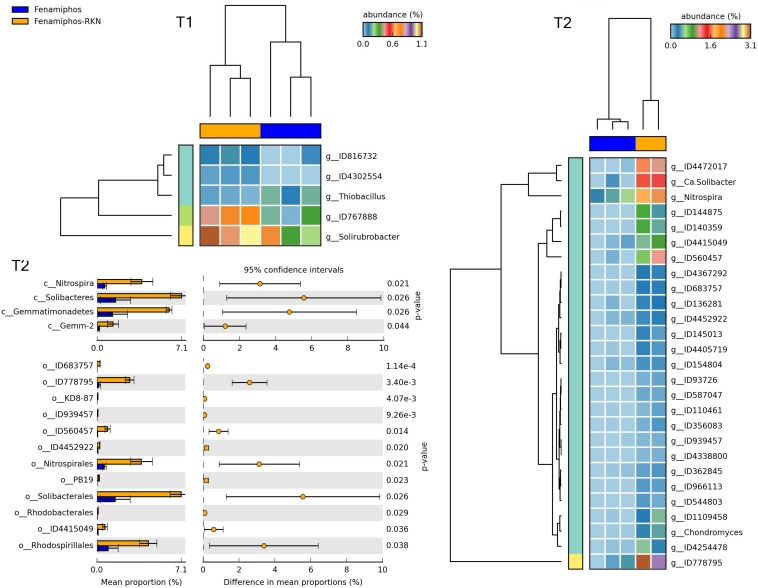
Heatmaps showing samples clustering by treatment with significant changes for abundance of genera (% of total; *P* ≤ 0.05; effect size, ratio of proportions = 0.8), when comparing samples of fenamiphos vs. fenamiphos with RKN inoculation, and significant changes in mean proportion (%) of sequences, at the class and order levels, at T_2_ (equal variance *t*-test, two sided; bars show SD).

**FIGURE 12:**
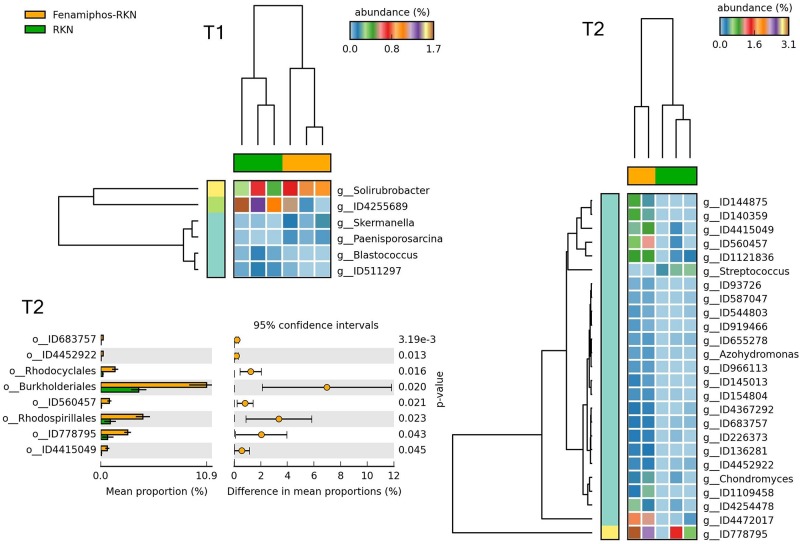
Heatmaps showing samples clustering by treatment with significant changes for abundance of genera (% of total; *P* ≤ 0.05; effect size, ratio of proportions = 0.8), when comparing samples of RKN inoculated samples vs. fenamiphos with RKN inoculation, and significant changes in mean proportion (%) of sequences, at the order level, at T_2_ (equal variance *t*-test, two sided; bars show SD).

When fenamiphos-treated samples differed for RKN inoculation, most differences were observed at T_2_ for classes Nitrospira, Solibacteres, Gemmatimonadetes and Gemm-2, with a higher number of genera differentially represented at T_2_, and different from those observed at T_1_ (Figure 11). Similarly, a higher number of differentially represented taxa were observed at T_2_ when comparing the samples with RKN, differing for addition of fenamiphos, with a higher representation for Burkholderiales and other orders at T_2_, including Rhodospirillales and the order of OTU ID778795 (Gemmatimonadetes) (Figure 12 and Supplementary Figure S5). Congruent clustering was also observed when comparing fenamiphos-treated samples with those inoculated with RKN, although for four genera only, whereas no difference was found at T_2_, apart of a higher relative abundance for *Streptococcus*, in the samples with nematodes (Supplementary Figure S6).

When comparing all samples pooled by treatment (*P* < 0.05; sequence filter maximum = 10; effect size filter as ratio of proportions = 0.8), a higher differential abundance was observed for *Streptococcus*, *Staphylococcus*, OTU ID134102 and others, in RKN samples *vs.* control. A detrimental effect of fenamiphos, in comparison to control, was found on *Lysobacter*, *Blastococcus* and OTU ID261264, with an increase for *Luteimonas* (Supplementary Figure S7). A higher number of taxa was differentially represented when comparing all controls to all samples with fenamiphos and RKN, including *Solirubrobacter*, *Afifella*, *Devosia*, and several other unclassified OTUs (Supplementary Figure S8).

Finally, four of the five genera reported as fenamiphos-degrading bacteria (Cabrera et al., 2010) were found, with low abundance, among the examined samples. At 6 months, *Cupriavidus* was found in four samples, one for each treatment. *Microbacterium* was present only in one sample with fenamiphos and nematode inoculum. *Sinorhizobium* occurred only in one sample treated with fenamiphos, whereas *Ralstonia* was found in a RKN-inoculated and a control sample.

## Discussion

Metabarcoding data showed changes in the bacterial abundance profiles related to the treatments applied to rhizosphere soil samples that had a common origin (a single site in an organic farm field). This result, not assumed *a priori*, was supported by quantitative data and appeared with different dataset profiles and mostly at lowest taxonomic depths. Morever, data indicated that the changes in soil bacterial abundance was mainly due to rare and unclassified taxa, in particular for the samples treated with fenamiphos and inoculated with RKN, as also shown by the Chao-1 index. Hence, the microbiome appeared sensitive to factors related to (or deriving by) the nematocide and/or the nematode additions, mostly operating at lowest and unclassified taxonomic levels, in particular after 6 months.

Rare taxa sensitivity to these drivers suggest that d chemical treatments and pest management may affect their abundance and, indirectly, the services these bacteria provide.

RKN and fenamiphos affected OTUs that were different at the two times, not intercepted nor available for sequencing in other treatments and/or at T_1_, because present at low initial numbers. The selective increase of specific taxa in the presence of nematodes (i.e., Nitrospirales, *Bacillus*, *Corynebacterium, Streptococcus*, *Staphylococcus*) may suggest an effect of novel substrates and/or of previously scarce molecules (i.e., chitin, polysaccharides) that increased due to the presence of J2, eggs and egg mass matrix. Similarly, also secondary metabolic by-products may have been involved in these changes. Some of these taxa, i.e., *Streptomyces* (Actinobacteria) were also found to be enriched in cysts of the soybean cyst nematode *Heterodera glycines* or in soil suppressive to *M. incognita* (Elhady et al., 2017; Hu et al., 2019). Other lineages that were enriched in cysts, i.e., Cytophagales (Bacteriodetes) were found at a doubled abundance in RKN and also in control samples at T_2_ (0.5 and 0.4% of total sequences, respectively), decreasing or remaining unchanged in the other treatments (Supplementary Figure S2). These data suggest a specificity in the lineages of nematode-associated bacteria, in part reflecting the soil type and/or the nematode species involved.

As the soil taxonomic metabarcoding profiles also changed by the sampling times, this effect was possibly integrated by the long-term activation and germination of durable, resting propagules, in particular for *Bacillus* spp. (Nicholson, 2002). This mechanism, however, occurred independently among treatments, but was mostly found in samples with addition of RKN and nematocide.

A second mechanism underpinning the shifts observed in the microbiome profiles appears linked to the biology of the organisms involved. The presence of *M. incognita* eggs and J2 likely influenced the abundance and dispersal of specific nematode-associated bacteria, through mechanisms such as passive transport onto the cuticle (Standing et al., 2006). Although the nematodes were surface sterilized before inoculation, the J2 possibly mobilized rare bacterial species associated to chitin or to damaged root tissues, as well as other soil organisms such as nematophagous fungi or protozoa and, indirectly, their associate microflora. The introduction of additional cuticle-associated durable endospores cannot be, furthermore, excluded. Similarly, we cannot exclude an effect due to the changes in root architecture, in particular as related to the formation of galls, an enhanced number of tips and the release of exudates by the stressed roots (Saleem et al., 2018; Hartman and Tringe, 2019).

RKN inoculation likely allowed also the introduction or mobilization of bacteria or endosymbionts associated to nematode oral region, gut and/or intestine (Noel and Atibalentja, 2006; Haegemann et al., 2009; Ladygina et al., 2009; Berg et al., 2016) or associated as endophytes in roots. All nematodes co-exist with specific bacterial groups in their environments (Poinar and Hansen, 1986), and their interactions also affect the surrounding soil structure and/or microbial communities (De Mesel et al., 2004). An enrichment, shown by taxonomic metabarcoding data in root endophytic bacteria was reported in RKN-parasitized tomato roots, with increased bacterial activities in galls related to polysaccharides degradation, carbohydrate/protein metabolism and nitrogen fixation (Tian et al., 2015). Finally, many bacteria are known to produce a variety of compounds such as antibiotics, nutrients, exoenzymes and signal molecules (Pierson and Pierson, 2007), affecting the density and composition of their surrounding microflora.

A partial effect of the nematocide application was observed at T1 with a significant increase in the root weight, and at T2 with a lower (although not significant) egg number (Supplementary Figure S4). The nematocide effect was, however, not evident when considering other variables, at both sampling times. This appeared related to fenamiphos half life (21 days), to the time of first nematocide application (1 month after initial RKN inoculation) and/or to its run off by irrigation water. The RKN persisted during the assay and no long-term extinction occurred at 6 months. This was likely the effect of a density dependent reaction, as the nematode populations rebound after a reduction caused by an external factor, i.e., a chemical treatment (Seinhorst, 1967). The nematocide effect was instead more visible in relation to the bacteria taxonomic metabarcoding profiles in the treated samples, also suggesting a possible effect of some degradation compounds.

In this study the organic farm soil was used to eliminate other factors, i.e., any effect related to previous applications of pesticides or residues, as occurs in conventional farming. A similar rationale was applied for RKN, so that soil was chosen also because of the absence of RKN, to avoid interference by already indigenous RKN populations. Most differences observed in bacteria diversity then reflected the RKN and nematicide treatments that were applied in the assay.

This study showed that adding further components to a soil food web likely alters one or more links established among functional groups, resulting in a complex of effects visible in time at the taxonomic metabarcoding scale. Similar considerations hold for the nematocide applied, considering its activity in relation to the presence of J2 cadavers, and its degradation and metabolism as well. Considering the cadavers, the J2 hatching, persistence in soil (usually 24–48 h when close to roots) and death are not synchronous, and a different experimental approach (i.e., time samplings at weekly or few days regular intervals) had be applied to test the contribution of cadavers to the observed changes in bacteria diversity. As concerns the nematocide degradation, although changes in OTUs abundance in presence of fenamiphos do not directly demonstrate a degrading capacity for taxa with higher abundance that were differentially represented in this treatment only, they may, however, reflect indirect effects due to the nematocide activity and/or changes in RKN numbers. Services of soil bacteria include also the metabolism of pesticides such as fenamiphos, including its degradation after long-term applications (Johnson, 1998; Cabrera et al., 2010).

Fenamiphos degradation by soil bacteria was shown in a wide range of soils with radio-labeled molecules, with varying recovery rates related to the soil types and temperature (Simon et al., 1992). Specialized soil bacteria degrading fenamiphos and its metabolites were identified by Cabrera et al. (2010). Other assays showed that a *Microbacterium esteraromaticum* isolate also degraded related toxic oxidation products such as fenamiphos sulfoxide and sulfone (Cáceres et al., 2008). Other fenamiphos-degrading species include a *Brevibacterium* sp., capable to rapidly hydrolyze a high fraction of the molecules adsorbed to an organo-clay complex (Singh et al., 2003; Singh and Walker, 2006). The insurgence of bacterial groups capable of degrading pesticides and related by-products was also observed for other active compounds, such as fenitrothion, whose introduction in soil increased *Burkholderia* spp. and other methylotrophs degrading its methanol derivative (Itoh et al., 2014). The degrading capacity of pesticides by soil bacteria represents a fundamental service, useful not only in case of pollution but also in sibling contamination issues such as the continuous agricultural practices. In the soil used in the assay there was a number of fenamiphos-degrading taxa that were found, however, at low abundance, suggesting either they did not increase because of the nematocide or that they had a transient effect, not revealed during the sampling intervals.

## Conclusion

The changes observed in the bacterial microbiome reflected abundance, reproduction and survival of species such as *Bacillus* spp., initially present at very low densities and increasing at 6 months in presence of RKN. Changes in relative abundance were observed at any taxonomic level and mostly involved unclassified OTUs, with a clustering of samples-by-treatments visible at lowest taxonomic levels. Sample clusterings indicated that the re-organization of the bacterial communities following treatments mainly concerned a range of specialized taxa, often belonging to distant lineages, shifting in time. Results also indicated a clear link of rhizosphere bacteria with phytoparasitic nematodes. Encompassing unclassified species, the data enlarge our knowledge on the functional role of rare or unculturable OTUs, as well as on their potential for nematode management.

## Data Availability Statement

All taxonomic metabarcoding datasets generated for this study are available at NCBI Bioproject PRJNA371772, https://www.ncbi.nlm.nih.gov/Traces/study/?acc=SRP099116.

## Author Contributions

AC, MC, and LS planned and designed the research. MC and LR performed the experiments. MC, AC, DC, and LR analyzed the data. AC and MC wrote the manuscript. All authors reviewed, revised, and approved the manuscript.

## Conflict of Interest

The authors declare that the research was conducted in the absence of any commercial or financial relationships that could be construed as a potential conflict of interest.

## Footnotes

1 https://github.com/neufeld/pandaseq

2 http://kiwi.cs.dal.ca/Software/STAMP

3 https://github.com/leffj/mctoolsr/

4 https://www.ncbi.nlm.nih.gov/Traces/study/?acc=SRP099116

## Supplementary Material

FIGURE S1 A schematic drawing **(a)** of the experimental design applied, showing the timing (in months) of chemical treatments (dots) and samplings for RNAseq and nematode analyses (arrows). OTUs genera counted in RNAseq data **(b)**, by treatment and sampling times. Plots of OTUs β-diversity by treatments **(c–e)**, as shown by Principal Coordinate Analysis (all samples, see colors in legend for treatments. Treatments codes: CON, untreated control; RKN, inoculated with *Meloidogyne incognita*; FEN, treated with fenamiphos; FEN-RKN, inoculated with *M. incognita* and fenamiphos).

FIGURE S2 Interactive ring-charts (html format) produced with Krona, showing the mean taxonomic repartitions and relative abundance of taxa resulting from the RNAseq analyses, by treatment and sampling times. For treatments codes see legend of Supplementary Figure S1. Files constructed using the mean of three replications, except CON at time T_0_ (prior to transplants), and FEN-RKN at T_2_ (6 months), with two replicates each. Unclassified taxa were retained in the analyses.

FIGURE S3 Venn diagrams showing the number of genera per treatment, at 3 (T_1_) and 6 months (T_2_). For control, the number of genera before planting (T_0_) is also shown.

FIGURE S4 Effect of treatments on *Meloidogyne incognita* egg density on roots **(A)**, root gall index (RGI, **B**), tomato root weight **(C)** and plant height **(D)**, 3 (T_1_) and 6 months (T_2_) after inoculation. Means ± SE from five replicates. Asterisks on bars show significant differences from corresponding controls at the same sampling time, as indicated by Student’s *t*-test, for *p* ≤ 0.05 (*) and *p* ≤ 0.01 (**). For treatments application see Supplementary Figure S1a.

FIGURE S5 Proportion of sequences (% of total) for selected OTUs at the three sampling times, by the treatments applied. Boxes show the quartile range (75th to 25th). The median is shown as a line within the box, a star indicates the mean. Whiskers indicate the most extreme data points within 1.5 ⋅ (75th – 25th percentile) of the median.

FIGURE S6 Differential representation and congruent clustering for significant changes found when comparing pooled samples for RKN and fenamiphos treated samples, at 3 months (T_1_). Differential abundance of *Streptococcus* 6 months (T_2_) after *Meloidogyne incognita* inoculation. Horizontal line across bars show the mean values.

FIGURE S7 Differential representation and congruent clustering for significant OTUs found in heatmaps and relative sequence proportions when comparing pooled samples for control vs. *Meloidogyne incognita* **(A)** and fenamiphos **(B)** (equal variance *t*-test, two sided; bars show SD).

FIGURE S8 Differential representation and clustering for significant OTUs shown as heatmap and relative sequence proportions when comparing pooled samples for control vs. fenamiphos with *Meloidogyne incognita* (equal variance *t*-test, two sided; bars show SD).

TABLE S1 Significant changes observed by sampling time for relative abundance of Bacteria (% of sequences), per treatment.

TABLE S2 Significant changes observed by sampling time for relative abundance of Bacteria (% of sequences) for all pooled samples and treatments.

TABLE S3 Significant changes observed for relative abundance of Bacteria (% of sequences) comparing control samples vs. samples treated with fenamiphos and inoculated with RKN.
