# Supplementary figures and images for: Response of Tomato Rhizosphere Bacteria to Root-Knot Nematodes, Fenamiphos and Sampling Time Shows Differential Effects on Low Level Taxa
Source: Front Microbiol. 2020 Mar 20;11:390. doi: 10.3389/fmicb.2020.00390 (PMC7100632; doi:10.3389/fmicb.2020.00390)

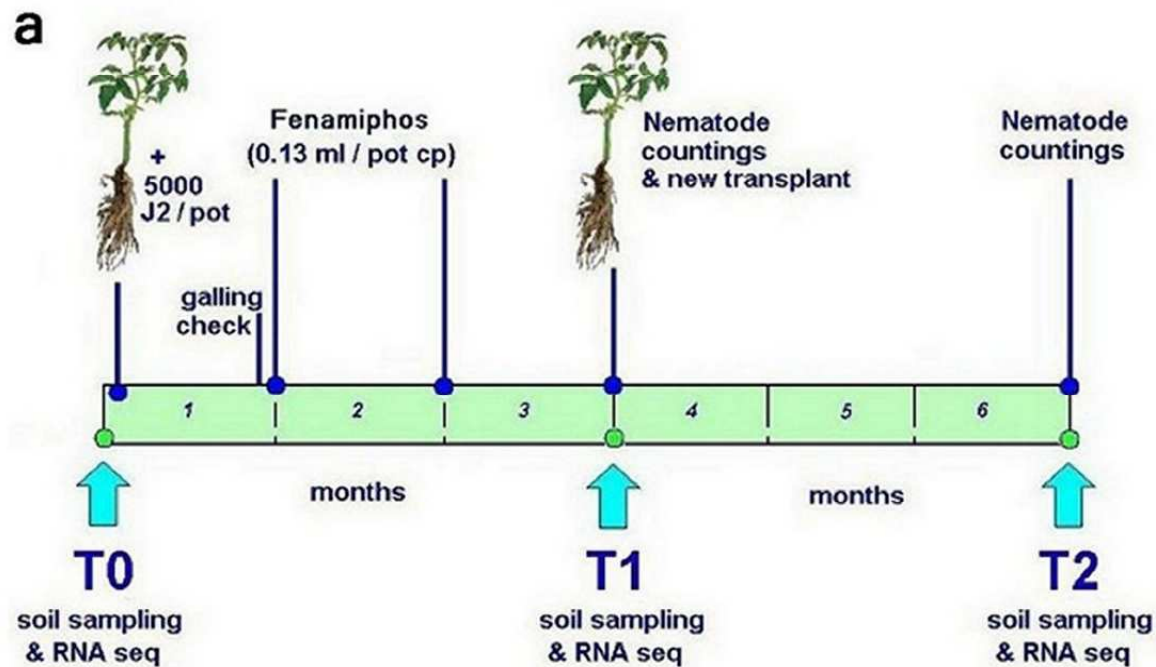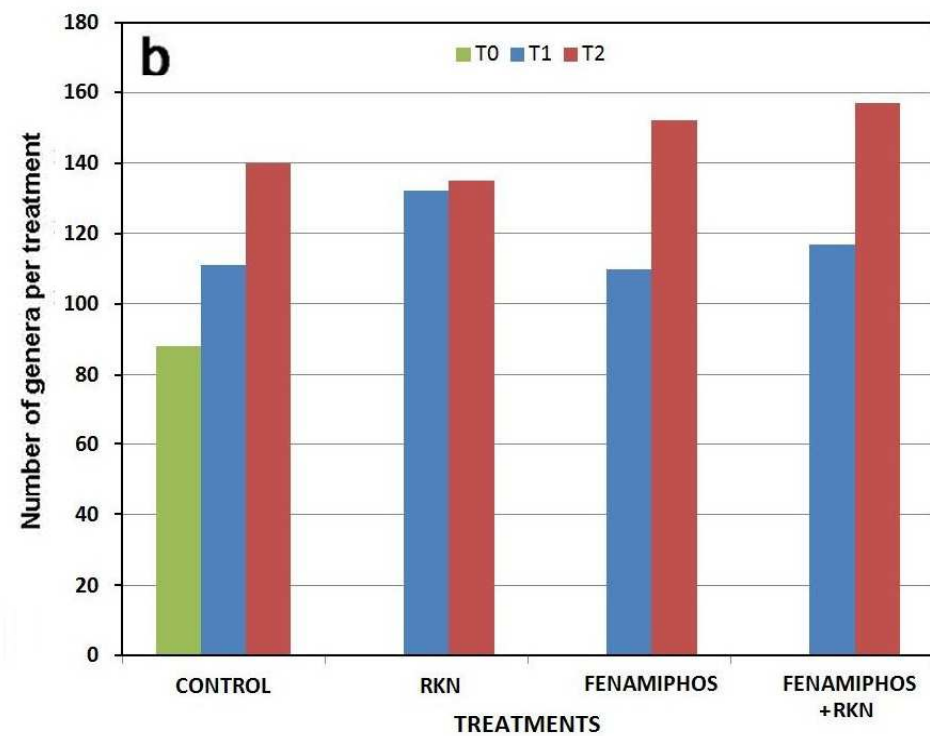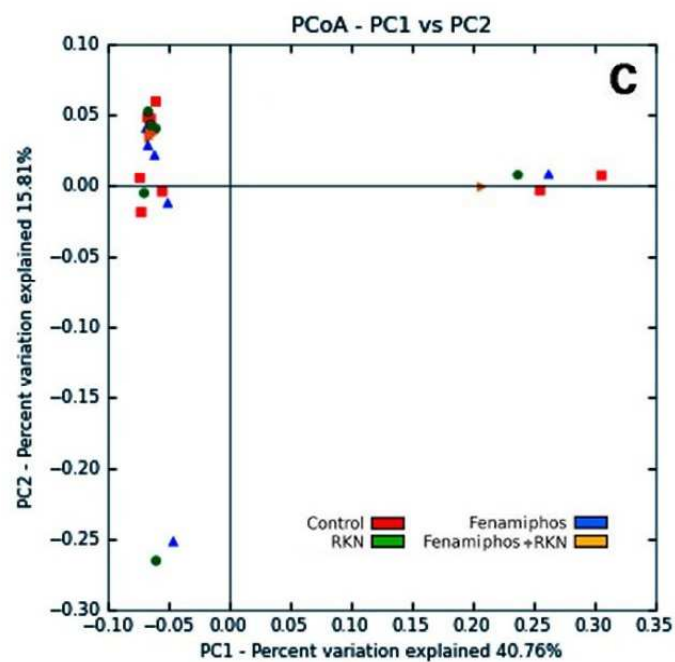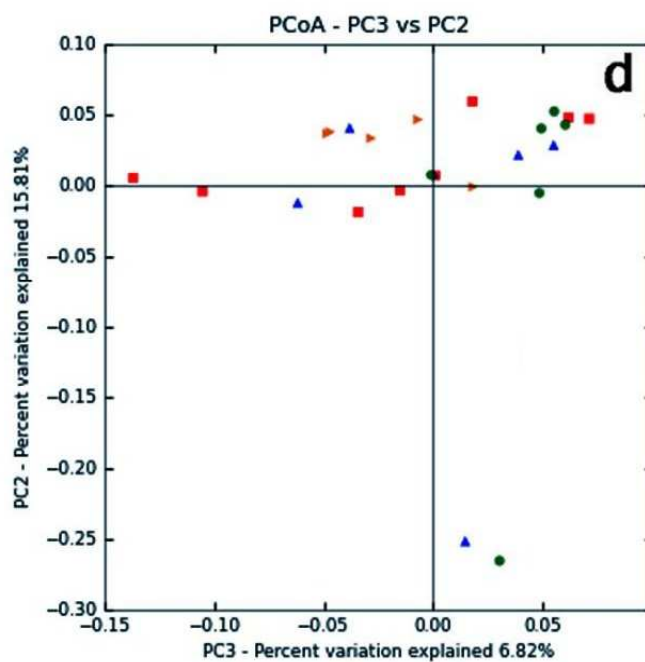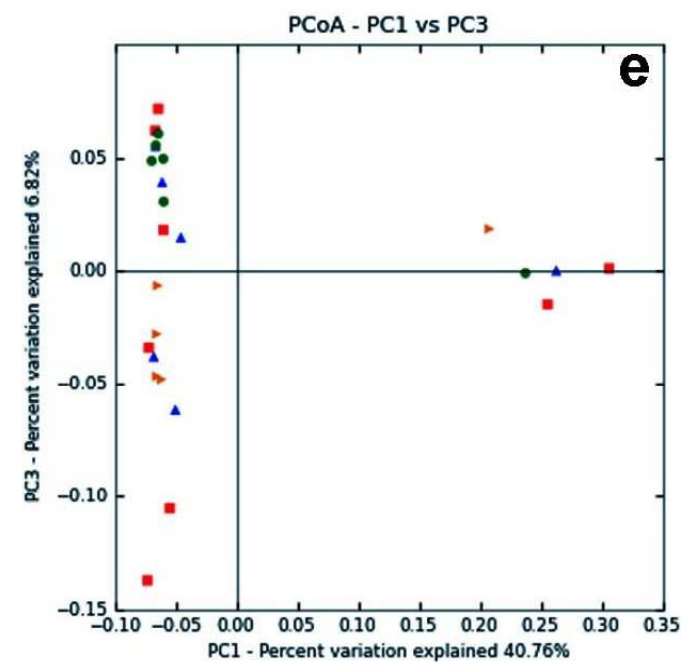

Supplement: FIGURE S1 — A schematic drawing (a) of the experimental design applied, showing the timing (in months) of chemical treatments (dots) and samplings for RNAseq and nematode analyses (arrows). OTUs genera counted in RNAseq data (b), by treatment and sampling times. Plots of OTUs β-diversity by treatments (c–e), as shown by Principal Coordinate Analysis (all samples, see colors in legend for treatments. Treatments codes: CON, untreated control; RKN, inoculated with Meloidogyne incognita; FEN, treated with fenamiphos; FEN-RKN, inoculated with M. incognita and fenamiphos). [file Presentation_1.PDF]

Proportion of sequences (%)

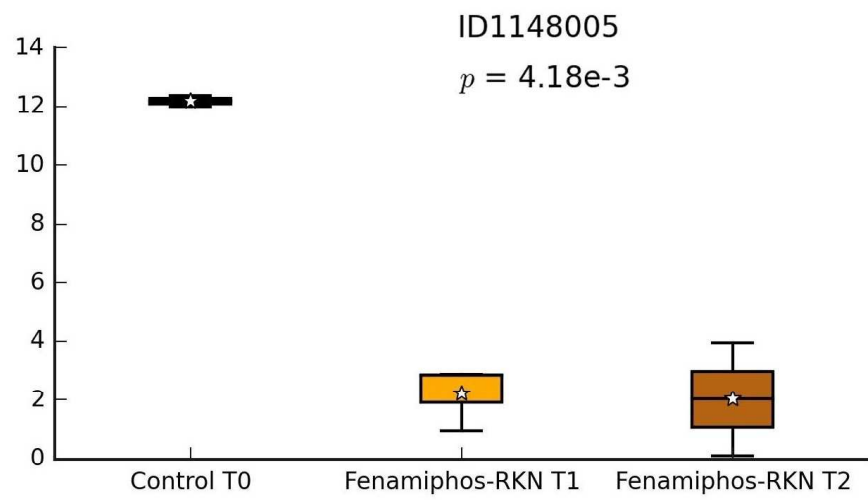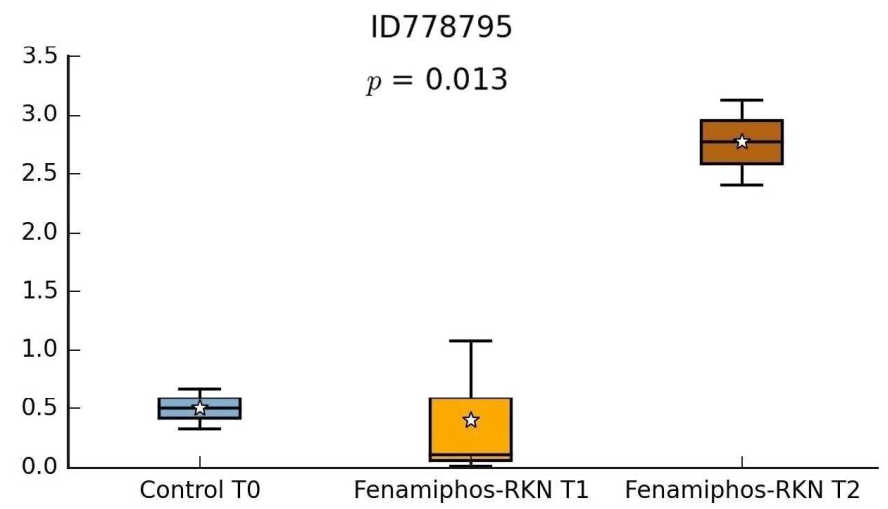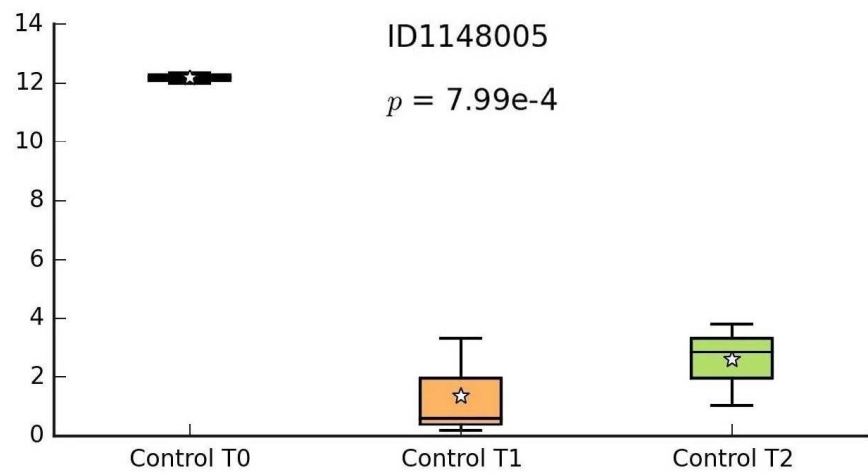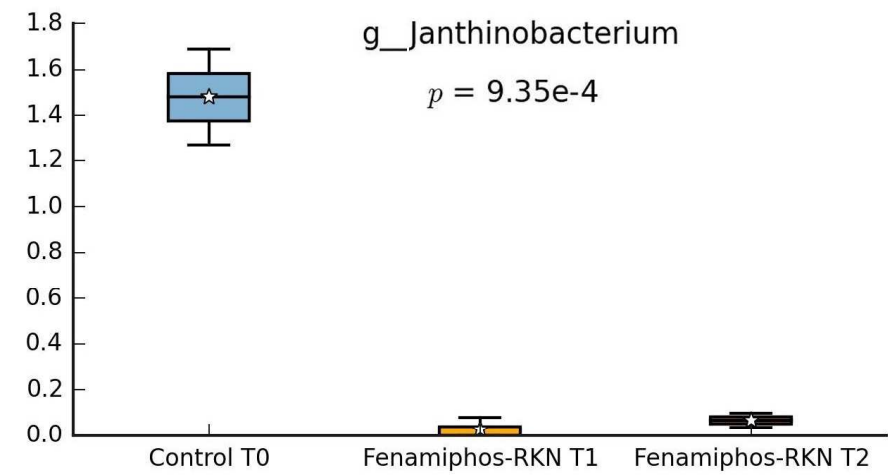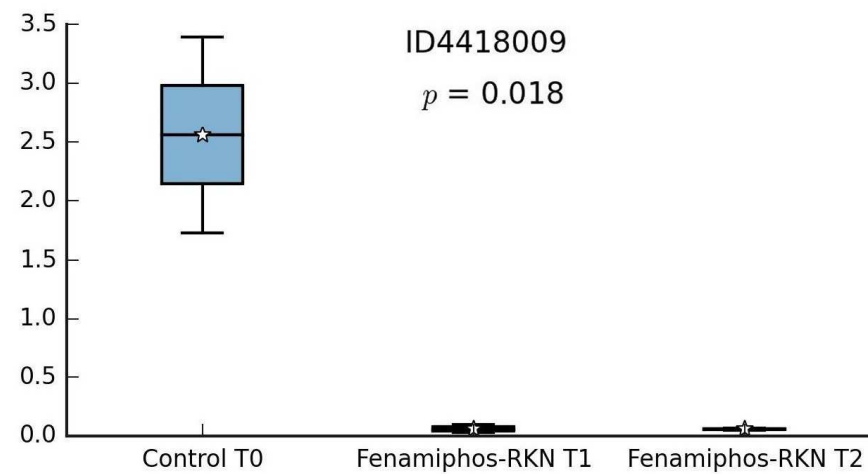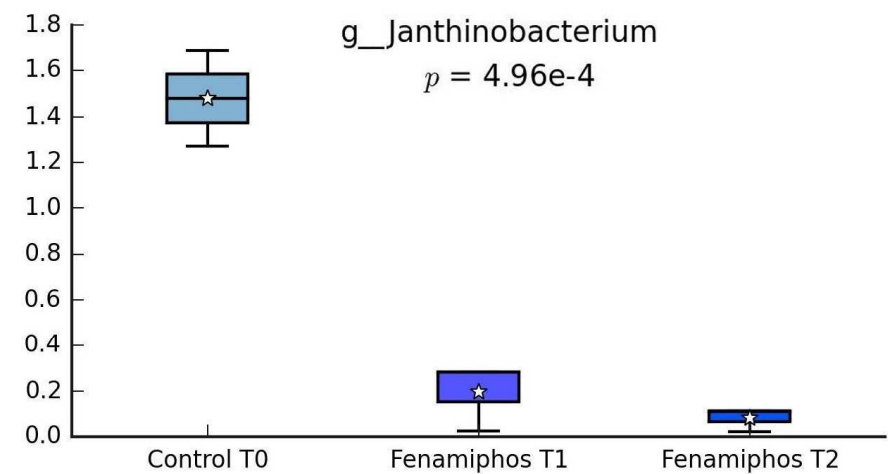

Supplement: FIGURE S4 — Effect of treatments on Meloidogyne incognita egg density on roots (A), root gall index (RGI, B), tomato root weight (C) and plant height (D), 3 (T1) and 6 months (T2) after inoculation. Means ± SE from five replicates. Asterisks on bars show significant differences from corresponding controls at the same sampling time, as indicated by Student’s t-test, for p ≤ 0.05 (*) and p ≤ 0.01 (**). For treatments application see Supplementary Figure S1a. [file Presentation_4.PDF]

Fenamiphos  
RKN

T1

T2

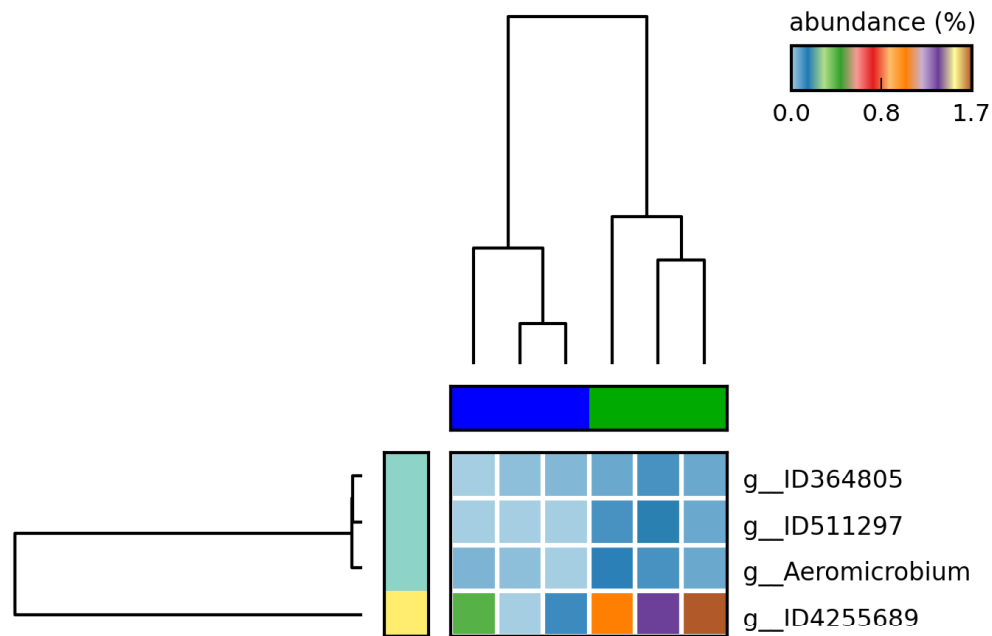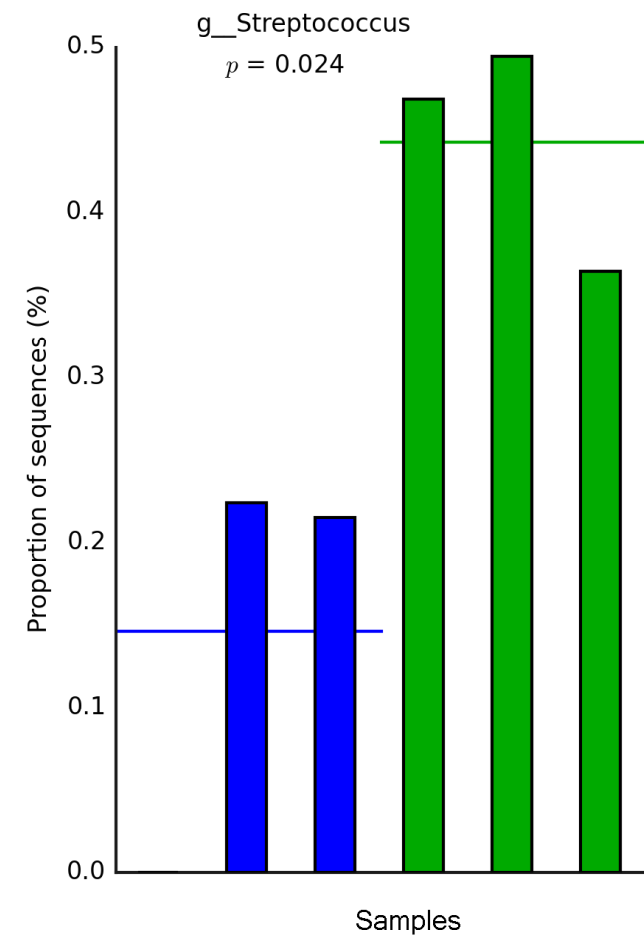

Supplement: FIGURE S5 — Proportion of sequences (% of total) for selected OTUs at the three sampling times, by the treatments applied. Boxes show the quartile range (75th to 25th). The median is shown as a line within the box, a star indicates the mean. Whiskers indicate the most extreme data points within 1.5 ⋅ (75th – 25th percentile) of the median. [file Presentation_5.PDF]

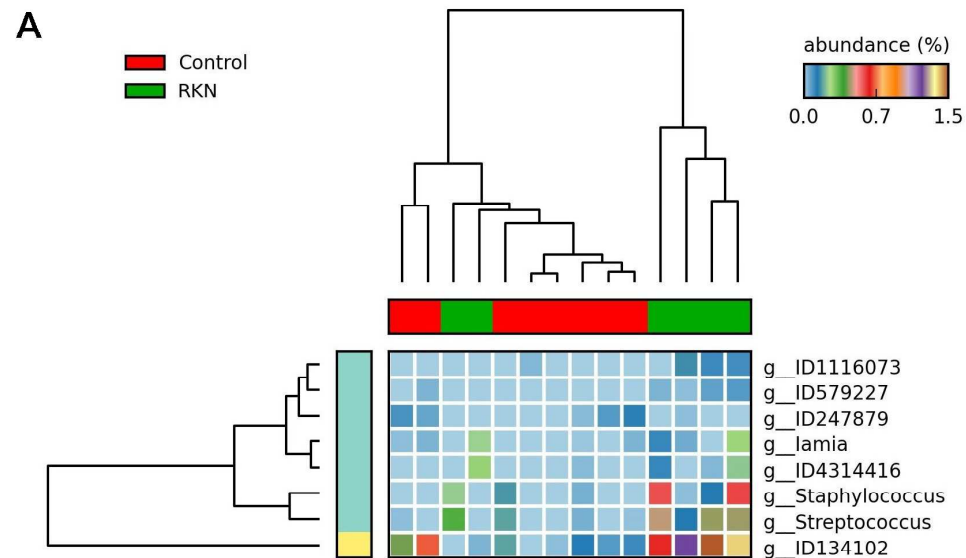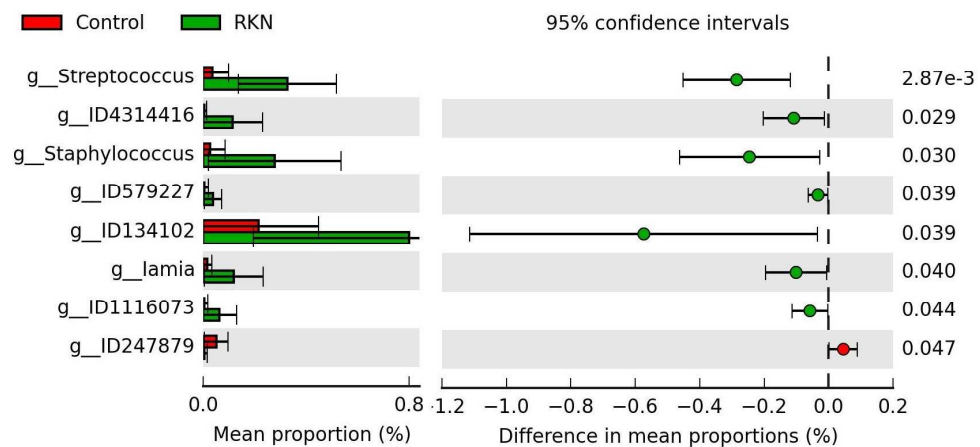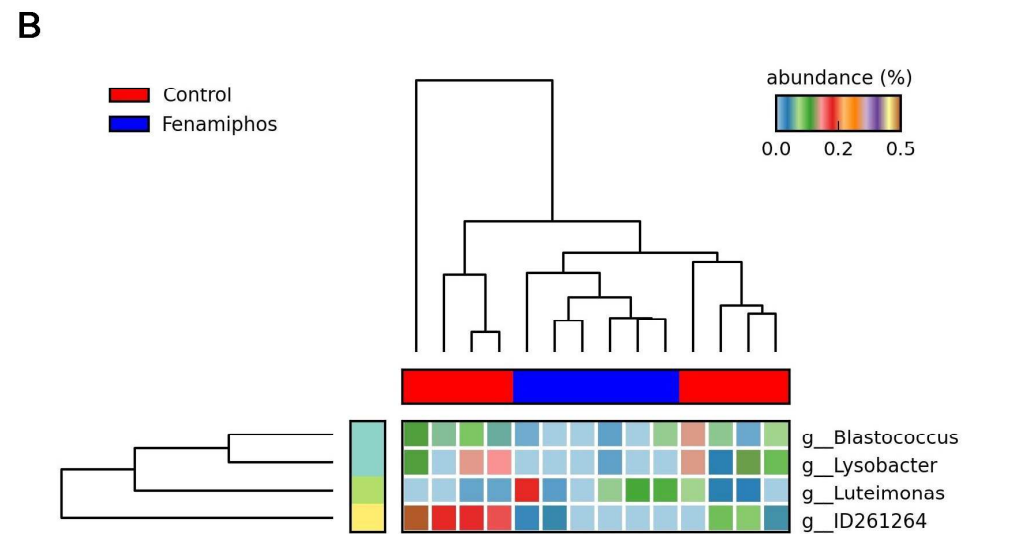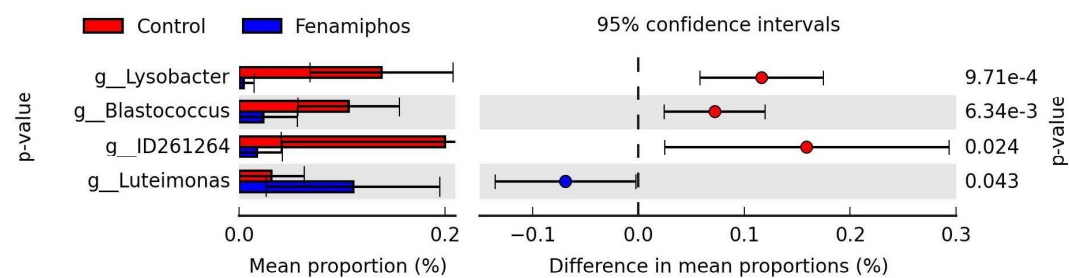

Supplement: FIGURE S6 — Differential representation and congruent clustering for significant changes found when comparing pooled samples for RKN and fenamiphos treated samples, at 3 months (T1). Differential abundance of Streptococcus 6 months (T2) after Meloidogyne incognita inoculation. Horizontal line across bars show the mean values. [file Presentation_6.PDF]

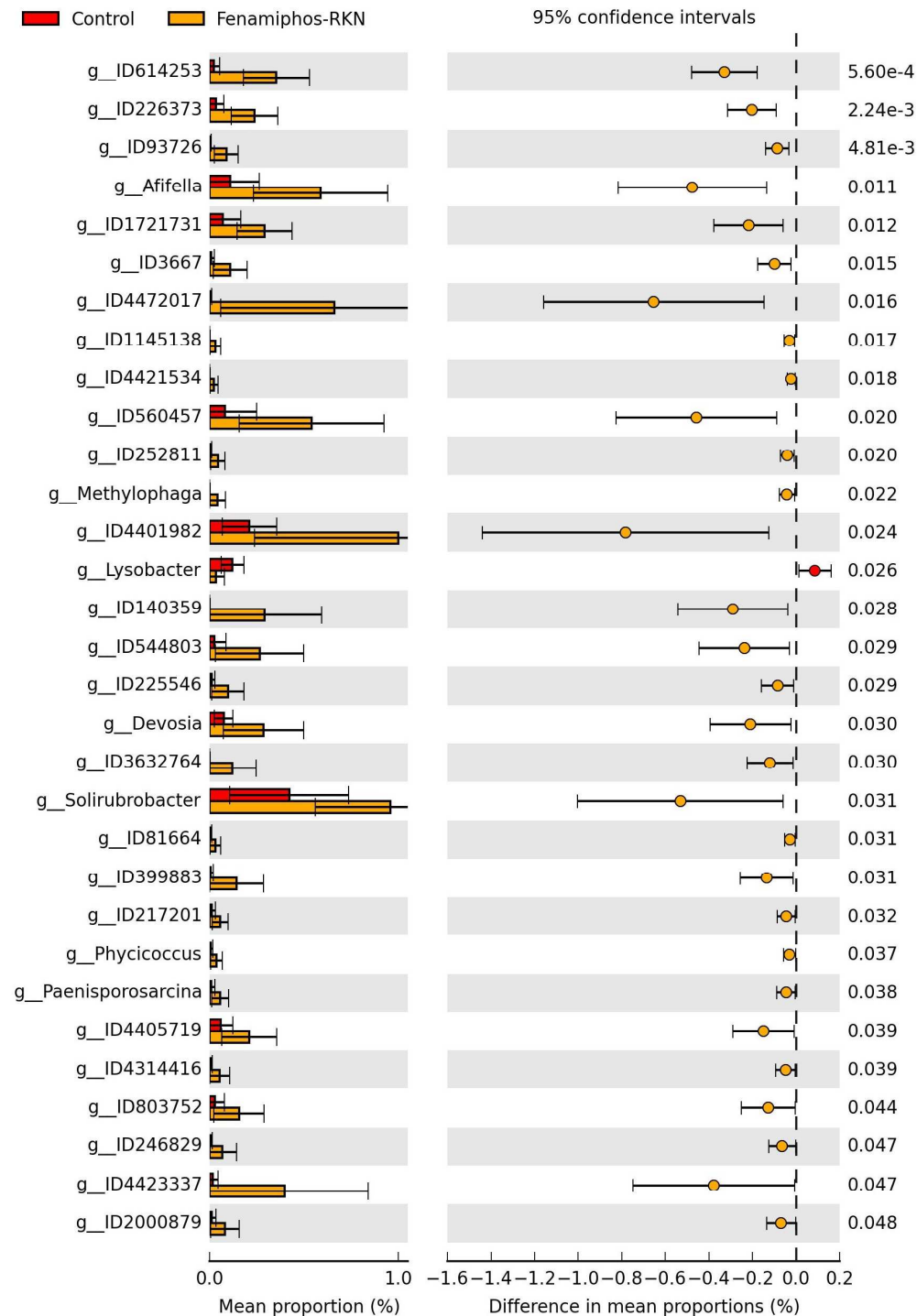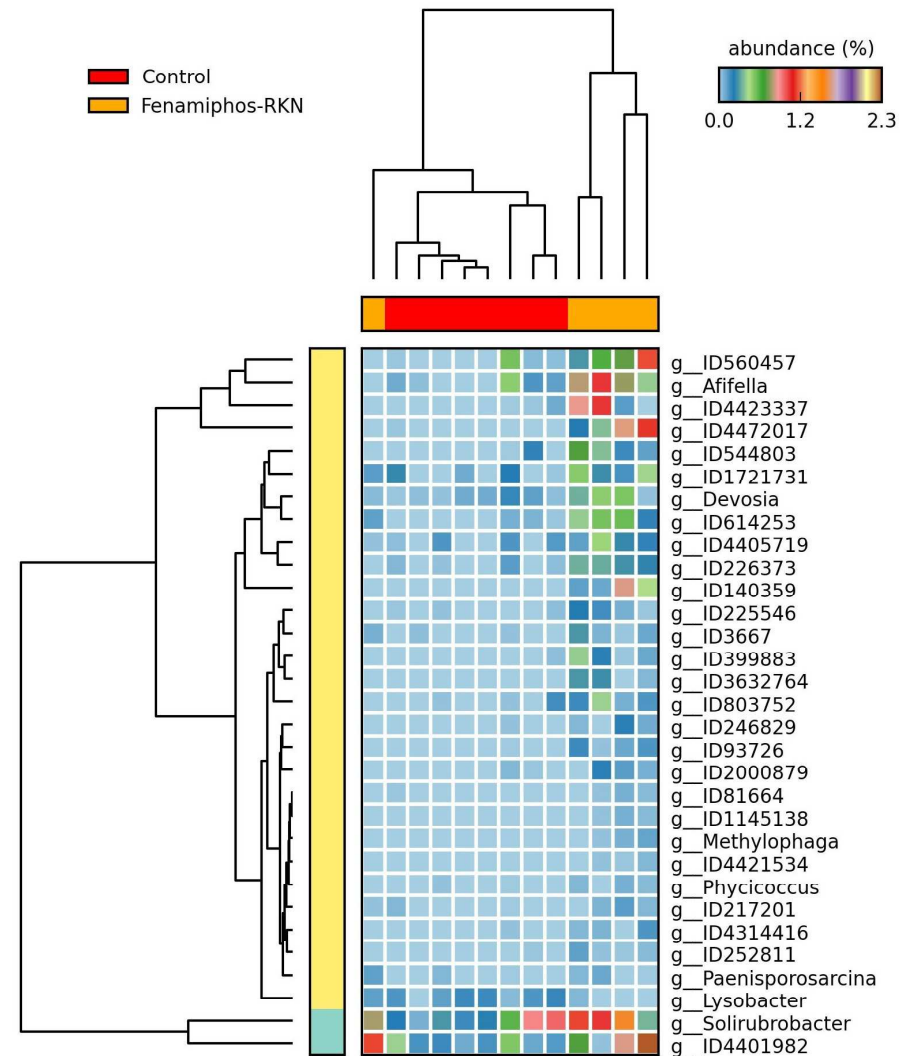

Supplement: FIGURE S7 — Differential representation and congruent clustering for significant OTUs found in heatmaps and relative sequence proportions when comparing pooled samples for control vs. Meloidogyne incognita (A) and fenamiphos (B) (equal variance t-test, two sided; bars show SD). [file Presentation_7.PDF]

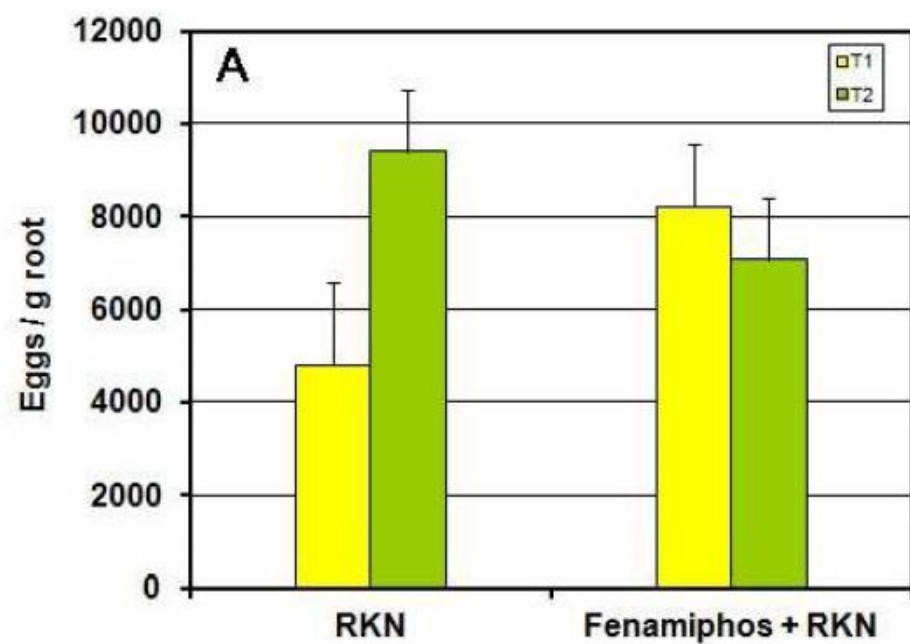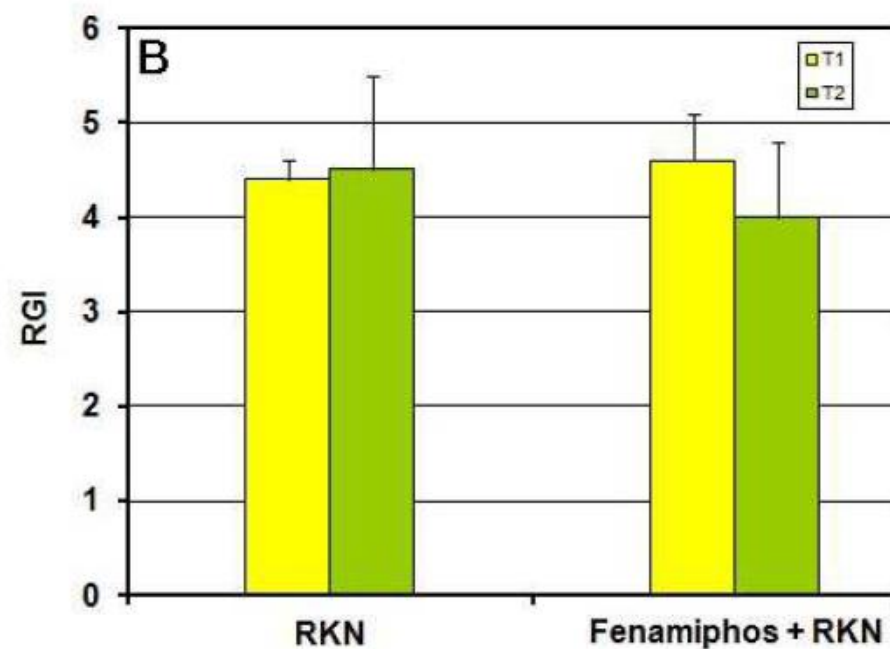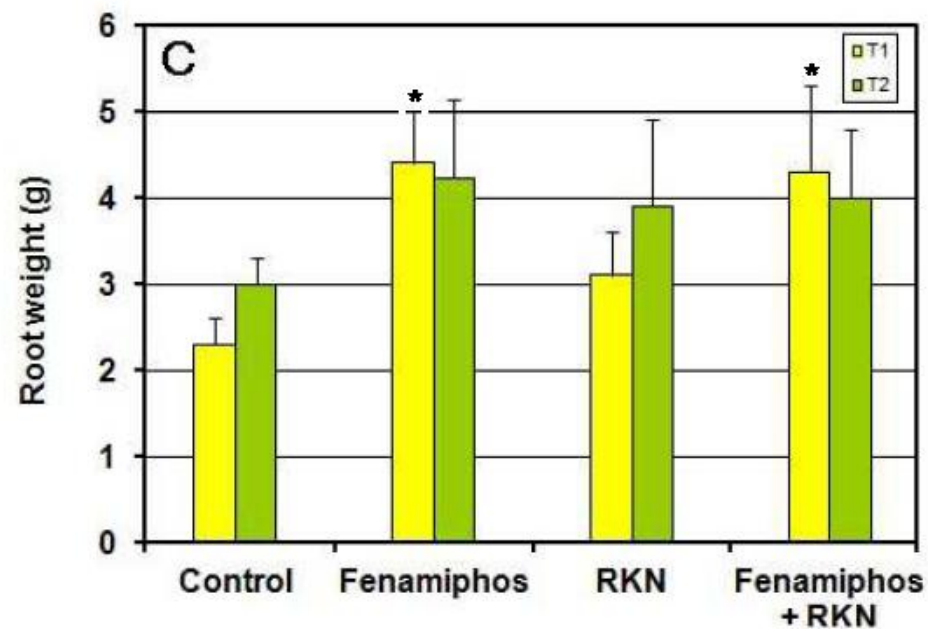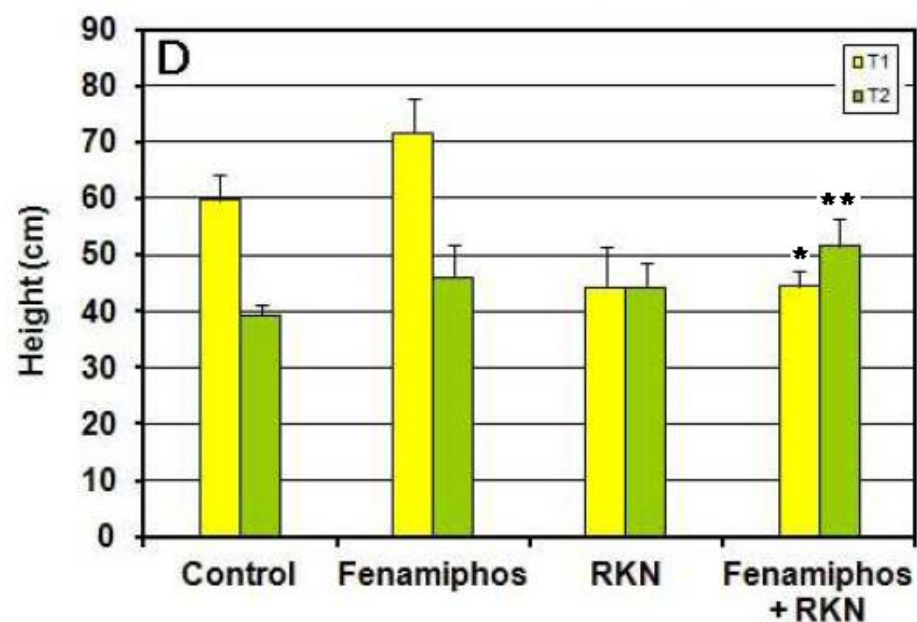

TREATMENTS

Supplement: FIGURE S8 — Differential representation and clustering for significant OTUs shown as heatmap and relative sequence proportions when comparing pooled samples for control vs. fenamiphos with Meloidogyne incognita (equal variance t-test, two sided; bars show SD). [file Data_Sheet_1.PDF]
