# Supplementary material for: Response of Tomato Rhizosphere Bacteria to Root-Knot Nematodes, Fenamiphos and Sampling Time Shows Differential Effects on Low Level Taxa
Source: Front Microbiol. 2020 Mar 20;11:390. doi: 10.3389/fmicb.2020.00390 (PMC7100632; doi:10.3389/fmicb.2020.00390)
Supplement: FIGURE S2 — Interactive ring-charts (html format) produced with Krona, showing the mean taxonomic repartitions and relative abundance of taxa resulting from the RNAseq analyses, by treatment and sampling times. For treatments codes see legend of Supplementary Figure S1. Files constructed using the mean of three replications, except CON at time T0 (prior to transplants), and FEN-RKN at T2 (6 months), with two replicates each. Unclassified taxa were retained in the analyses. [file Presentation_2.zip › CONTROL T0 mean.html]

Javascript must be enabled to view this page.

magnitude
 3015.5
 3015.5
 37
 37
 37
 34
 10.5
 23.5
 3
 3
 402.5
 120
 120
 15
 15
 18.5
 16.5
 1.5
 .5
 24
 15.5
 8.5
 6
 2.5
 .5
 1.5
 1.5
 15
 12
 1.5
 .5
 1
 4
 1
 3
 7.5
 1
 1
 1.5
 4
 17.5
 16
 1.5
 .5
 .5
 2
 .5
 1.5
 4
 2
 2
 2
 2
 2.5
 2.5
 .5
 .5
 .5
 .5
 .5
 .5
 70
 70
 68.5
 68.5
 1
 1
 .5
 .5
 56
 52.5
 22
 16
 6
 20
 20
 4
 4
 6.5
 6.5
 3.5
 3
 3
 .5
 .5
 1.5
 1.5
 1.5
 1.5
 153
 153
 153
 152.5
 .5
 2
 2
 2
 2
 1983
 184
 115
 2.5
 1
 .5
 .5
 .5
 28.5
 27.5
 1
 2
 2
 2.5
 2
 .5
 1
 .5
 .5
 2.5
 2.5
 68
 68
 6.5
 1
 5.5
 1.5
 1.5
 4
 3.5
 .5
 2
 1
 .5
 .5
 14
 12.5
 3
 .5
 8.5
 .5
 1.5
 1.5
 45.5
 44.5
 43.5
 1
 1
 1
 .5
 .5
 .5
 1.5
 1.5
 1.5
 3.5
 2
 2
 1.5
 1.5
 1527
 31.5
 31.5
 31.5
 636
 221.5
 162.5
 .5
 1
 5
 3.5
 4
 .5
 1.5
 .5
 1.5
 .5
 1.5
 32
 2
 5
 406
 356.5
 .5
 .5
 48.5
 6
 6
 2.5
 1.5
 1
 498.5
 498.5
 498.5
 316
 316
 315.5
 .5
 5.5
 5.5
 5.5
 7
 7
 6.5
 .5
 7.5
 7.5
 6
 1.5
 2.5
 2.5
 2.5
 4.5
 4.5
 4.5
 .5
 .5
 .5
 17.5
 17.5
 17.5
 168
 4.5
 3.5
 3.5
 1
 1
 155.5
 115
 115
 3
 3
 2.5
 2
 .5
 .5
 .5
 34.5
 34.5
 6
 6
 4.5
 1.5
 .5
 .5
 .5
 1.5
 1.5
 1.5
 104
 99.5
 83
 7
 60
 1.5
 4.5
 10
 16.5
 5.5
 11
 1
 1
 .5
 .5
 .5
 .5
 .5
 1.5
 1.5
 1.5
 1.5
 1.5
 1.5
 460.5
 451
 451
 1.5
 1.5
 95.5
 95
 .5
 353
 353
 1
 1
 5
 5
 5
 5
 1
 1
 1
 1
 1.5
 1.5
 1.5
 1.5
 2
 2
 2
 .5
 1.5
 63.5
 9.5
 9.5
 9.5
 9.5
 38.5
 25
 25
 25
 9.5
 2
 2
 7
 7
 .5
 .5
 .5
 .5
 .5
 3.5
 3.5
 3.5
 2.5
 2.5
 2.5
 2.5
 5.5
 5.5
 5.5
 5.5
 4.5
 4.5
 4.5
 4.5
 3
 3
 3
 3
 7
 1
 1
 1
 1
 1
 1
 1
 1
 .5
 .5
 .5
 .5
 1
 .5
 .5
 .5
 .5
 .5
 .5
 .5
 .5
 .5
 .5
 .5
 .5
 .5
 .5
 2.5
 1
 1
 1
 1.5
 1.5
 1.5
 34.5
 34.5
 34
 24.5
 20.5
 4
 5.5
 2
 3.5
 2.5
 2.5
 1.5
 .5
 .5
 .5
 .5
 .5
 .5
 6.5
 .5
 .5
 .5
 .5
 .5
 .5
 .5
 .5
 3
 3
 3
 3
 2.5
 2.5
 2.5
 2.5
 .5
 .5
 .5
 .5
 .5
 1.5
 1.5
 1.5
 1.5
 1.5
 1
 1
 1
 1
 1
 1.5
 .5
 .5
 .5
 .5
 1
 1
 1
 1
 4
 4
 4
 4
 4
 .5
 .5
 .5
 .5
 .5
 4.5
 3
 3
 3
 1
 2
 1.5
 1.5
 1.5
 1.5
 7.5
 7.5
 7.5
 7.5
 7.5
