# Supplementary material for: Response of Tomato Rhizosphere Bacteria to Root-Knot Nematodes, Fenamiphos and Sampling Time Shows Differential Effects on Low Level Taxa
Source: Front Microbiol. 2020 Mar 20;11:390. doi: 10.3389/fmicb.2020.00390 (PMC7100632; doi:10.3389/fmicb.2020.00390)
Supplement: FIGURE S2 — Interactive ring-charts (html format) produced with Krona, showing the mean taxonomic repartitions and relative abundance of taxa resulting from the RNAseq analyses, by treatment and sampling times. For treatments codes see legend of Supplementary Figure S1. Files constructed using the mean of three replications, except CON at time T0 (prior to transplants), and FEN-RKN at T2 (6 months), with two replicates each. Unclassified taxa were retained in the analyses. [file Presentation_2.zip › CONTROL T1 mean.html]

Javascript must be enabled to view this page.

magnitude
 3894.33333333333
 3566.33333333333
 1393.33333333333
 1
 1
 1
 1
 64.3333333333333
 64.3333333333333
 62
 62
 .666666666666667
 .666666666666667
 1.33333333333333
 1.33333333333333
 .333333333333333
 .333333333333333
 688.666666666667
 661.333333333333
 603.666666666667
 603.666666666667
 46.3333333333333
 29.3333333333333
 17
 10.3333333333333
 10.3333333333333
 1
 1
 27.3333333333333
 27.3333333333333
 27.3333333333333
 619
 617.333333333333
 109.333333333333
 79.3333333333333
 2.66666666666667
 8.66666666666667
 11.3333333333333
 7.33333333333333
 22.6666666666667
 5.66666666666667
 1.66666666666667
 11
 4.33333333333333
 5.33333333333333
 3
 .333333333333333
 1.33333333333333
 .333333333333333
 .333333333333333
 135.666666666667
 86
 46
 .666666666666667
 .666666666666667
 2.33333333333333
 162.333333333333
 162.333333333333
 23
 17
 .333333333333333
 4
 1.66666666666667
 1.33333333333333
 .333333333333333
 .333333333333333
 .666666666666667
 5.33333333333333
 3
 .333333333333333
 .333333333333333
 .333333333333333
 .333333333333333
 1
 61.3333333333333
 16.3333333333333
 45
 3
 .333333333333333
 1.33333333333333
 1.33333333333333
 2.33333333333333
 .666666666666667
 .333333333333333
 1.33333333333333
 15
 15
 .333333333333333
 .333333333333333
 .333333333333333
 .333333333333333
 1
 1
 5
 5
 45.3333333333333
 45.3333333333333
 .333333333333333
 .333333333333333
 2
 .333333333333333
 1.66666666666667
 .333333333333333
 .333333333333333
 2
 2
 14
 1.33333333333333
 12.6666666666667
 1.66666666666667
 1.66666666666667
 1.66666666666667
 20.3333333333333
 20.3333333333333
 20.3333333333333
 1.33333333333333
 19
 202.333333333333
 127.333333333333
 127.333333333333
 96.6666666666667
 96.6666666666667
 29
 29
 1.66666666666667
 1.66666666666667
 14.3333333333333
 13.3333333333333
 13.3333333333333
 13.3333333333333
 1
 1
 1
 51.6666666666667
 50.6666666666667
 47
 47
 3.66666666666667
 3.66666666666667
 .333333333333333
 .333333333333333
 .333333333333333
 .666666666666667
 .666666666666667
 .666666666666667
 6.66666666666667
 6.66666666666667
 6.66666666666667
 6.66666666666667
 .333333333333333
 .333333333333333
 .333333333333333
 .333333333333333
 2
 2
 2
 2
 1621.66666666667
 198.666666666667
 167
 88.3333333333333
 74.6666666666667
 4.66666666666667
 .333333333333333
 1
 1.66666666666667
 .333333333333333
 4.33333333333333
 1.33333333333333
 78.6666666666667
 20.3333333333333
 58.3333333333333
 15.6666666666667
 .666666666666667
 .666666666666667
 .333333333333333
 .333333333333333
 14.6666666666667
 14.6666666666667
 1.66666666666667
 1.66666666666667
 1.66666666666667
 6
 5.66666666666667
 4
 1.66666666666667
 .333333333333333
 .333333333333333
 .333333333333333
 .333333333333333
 .333333333333333
 5.33333333333333
 5.33333333333333
 5.33333333333333
 2.66666666666667
 2.66666666666667
 2.66666666666667
 1198
 908.666666666667
 872.666666666667
 679.333333333333
 193.333333333333
 31.6666666666667
 1.33333333333333
 21.6666666666667
 1
 .333333333333333
 .666666666666667
 1
 .333333333333333
 1
 1.33333333333333
 3
 4.33333333333333
 4.33333333333333
 59
 59
 50.6666666666667
 8.33333333333333
 3
 3
 2.66666666666667
 .333333333333333
 .333333333333333
 .333333333333333
 .333333333333333
 1
 1
 1
 218
 218
 218
 .333333333333333
 .333333333333333
 .333333333333333
 1
 1
 .333333333333333
 .666666666666667
 .333333333333333
 .333333333333333
 .333333333333333
 3.33333333333333
 3.33333333333333
 3.33333333333333
 2
 2
 2
 1
 1
 1
 .333333333333333
 .333333333333333
 .333333333333333
 .333333333333333
 74
 50.6666666666667
 36
 36
 1
 1
 3.66666666666667
 3.66666666666667
 6.66666666666667
 6.66666666666667
 3.33333333333333
 2.33333333333333
 1
 .333333333333333
 .333333333333333
 .333333333333333
 18.6666666666667
 18.6666666666667
 18.6666666666667
 2
 .333333333333333
 .333333333333333
 1.66666666666667
 1.66666666666667
 2.33333333333333
 2.33333333333333
 2.33333333333333
 150.666666666667
 64
 .333333333333333
 .333333333333333
 3.66666666666667
 3.33333333333333
 .333333333333333
 7.66666666666667
 2
 5.66666666666667
 2.33333333333333
 2.33333333333333
 6.66666666666667
 .333333333333333
 6.33333333333333
 6
 6
 35
 35
 2.33333333333333
 2.33333333333333
 67.6666666666667
 55.6666666666667
 54.6666666666667
 .666666666666667
 .333333333333333
 9.33333333333333
 9.33333333333333
 2.66666666666667
 2.66666666666667
 12
 10
 3.33333333333333
 5.66666666666667
 1
 2
 2
 3.66666666666667
 3.66666666666667
 3.66666666666667
 1.33333333333333
 1.33333333333333
 1.33333333333333
 .333333333333333
 .333333333333333
 .333333333333333
 1.66666666666667
 1.66666666666667
 1.66666666666667
 30
 28.3333333333333
 25
 7
 5.33333333333333
 1.66666666666667
 .666666666666667
 .666666666666667
 2.66666666666667
 2.66666666666667
 1
 1
 2.66666666666667
 2.66666666666667
 11
 1.33333333333333
 4
 5.33333333333333
 .333333333333333
 3.33333333333333
 3.33333333333333
 3.33333333333333
 1.66666666666667
 1.66666666666667
 .333333333333333
 .333333333333333
 .333333333333333
 .333333333333333
 1
 1
 166
 84.6666666666667
 63
 63
 63
 13
 10
 10
 1.66666666666667
 1.66666666666667
 1.33333333333333
 1.33333333333333
 2.33333333333333
 2.33333333333333
 2.33333333333333
 2
 2
 2
 4.33333333333333
 4.33333333333333
 4.33333333333333
 14.6666666666667
 14.6666666666667
 14.6666666666667
 14.6666666666667
 13.6666666666667
 13.6666666666667
 13.6666666666667
 13.6666666666667
 40.3333333333333
 40.3333333333333
 40.3333333333333
 40.3333333333333
 3.33333333333333
 3.33333333333333
 3.33333333333333
 3.33333333333333
 9.33333333333333
 9.33333333333333
 9.33333333333333
 9.33333333333333
 11.6666666666667
 11
 2.33333333333333
 2.33333333333333
 2
 .333333333333333
 8
 7.33333333333333
 7.33333333333333
 .666666666666667
 .666666666666667
 .666666666666667
 .666666666666667
 .666666666666667
 .666666666666667
 .666666666666667
 .666666666666667
 .666666666666667
 4.33333333333333
 2.66666666666667
 2.66666666666667
 2.66666666666667
 2.66666666666667
 .333333333333333
 .333333333333333
 .333333333333333
 .333333333333333
 1.33333333333333
 1.33333333333333
 .333333333333333
 .333333333333333
 1
 .333333333333333
 .666666666666667
 16.3333333333333
 5.66666666666667
 5.66666666666667
 4.66666666666667
 1.66666666666667
 .666666666666667
 2.33333333333333
 1
 1
 8.66666666666667
 8.66666666666667
 7
 .666666666666667
 4
 1.33333333333333
 1
 1.66666666666667
 1.66666666666667
 1.33333333333333
 1.33333333333333
 1.33333333333333
 1.33333333333333
 .666666666666667
 .666666666666667
 .666666666666667
 .666666666666667
 5
 2.33333333333333
 2.33333333333333
 2.33333333333333
 2.33333333333333
 1.66666666666667
 1.66666666666667
 1.66666666666667
 .333333333333333
 1.33333333333333
 .333333333333333
 .333333333333333
 .333333333333333
 .333333333333333
 .666666666666667
 .666666666666667
 .666666666666667
 .666666666666667
 60.6666666666667
 15.3333333333333
 15.3333333333333
 15.3333333333333
 15.3333333333333
 1
 1
 1
 1
 23
 1.66666666666667
 1.66666666666667
 1.66666666666667
 .333333333333333
 .333333333333333
 .333333333333333
 21
 21
 21
 2.66666666666667
 .666666666666667
 .666666666666667
 .666666666666667
 .333333333333333
 .333333333333333
 .333333333333333
 1.66666666666667
 1.66666666666667
 1.66666666666667
 1.66666666666667
 .666666666666667
 .666666666666667
 .666666666666667
 .333333333333333
 .333333333333333
 .333333333333333
 .666666666666667
 .666666666666667
 .666666666666667
 1.66666666666667
 1.66666666666667
 1.66666666666667
 1.66666666666667
 11.3333333333333
 2.33333333333333
 2.33333333333333
 2.33333333333333
 9
 9
 9
 4
 4
 4
 4
 51.6666666666667
 51.6666666666667
 51.6666666666667
 20
 20
 31.6666666666667
 6
 25.6666666666667
 .333333333333333
 .333333333333333
 .333333333333333
 .333333333333333
 .333333333333333
 .333333333333333
 .333333333333333
 .333333333333333
 .333333333333333
 .333333333333333
 2.66666666666667
 2.66666666666667
 2.66666666666667
 2.66666666666667
 2.66666666666667
 328
 328
 328
 328
 328
 245
 83
