# Supplementary material for: Response of Tomato Rhizosphere Bacteria to Root-Knot Nematodes, Fenamiphos and Sampling Time Shows Differential Effects on Low Level Taxa
Source: Front Microbiol. 2020 Mar 20;11:390. doi: 10.3389/fmicb.2020.00390 (PMC7100632; doi:10.3389/fmicb.2020.00390)
Supplement: FIGURE S2 — Interactive ring-charts (html format) produced with Krona, showing the mean taxonomic repartitions and relative abundance of taxa resulting from the RNAseq analyses, by treatment and sampling times. For treatments codes see legend of Supplementary Figure S1. Files constructed using the mean of three replications, except CON at time T0 (prior to transplants), and FEN-RKN at T2 (6 months), with two replicates each. Unclassified taxa were retained in the analyses. [file Presentation_2.zip › CONTROL T2 mean.html]

Javascript must be enabled to view this page.

magnitude
 4797.66666666667
 4595.66666666667
 1457.33333333333
 209.333333333333
 209.333333333333
 199.666666666667
 199.666666666667
 1
 1
 2
 2
 .333333333333333
 .333333333333333
 5.33333333333333
 5.33333333333333
 1
 1
 553
 551.333333333333
 3
 3
 166.666666666667
 68.3333333333333
 94.6666666666667
 1.33333333333333
 .333333333333333
 2
 88
 88
 12.6666666666667
 9.33333333333333
 .333333333333333
 .333333333333333
 .666666666666667
 .333333333333333
 .333333333333333
 .666666666666667
 .666666666666667
 94.6666666666667
 82.6666666666667
 5.33333333333333
 3.66666666666667
 3
 7.33333333333333
 4
 .666666666666667
 .666666666666667
 2
 11
 8.33333333333333
 .333333333333333
 2.33333333333333
 13
 13
 6.66666666666667
 5.33333333333333
 .333333333333333
 1
 47.3333333333333
 38.6666666666667
 8.66666666666667
 8
 .666666666666667
 .333333333333333
 7
 16
 .666666666666667
 .333333333333333
 3.33333333333333
 10.3333333333333
 1.33333333333333
 58.6666666666667
 40
 2.66666666666667
 12.3333333333333
 3.66666666666667
 .333333333333333
 .333333333333333
 3.66666666666667
 3
 .666666666666667
 3.33333333333333
 1.33333333333333
 2
 .333333333333333
 .333333333333333
 .666666666666667
 .666666666666667
 2.66666666666667
 .333333333333333
 2.33333333333333
 .333333333333333
 .333333333333333
 3.66666666666667
 3.66666666666667
 .666666666666667
 .666666666666667
 .666666666666667
 .666666666666667
 1
 1
 1
 1
 1.66666666666667
 1.66666666666667
 1.66666666666667
 4.66666666666667
 .333333333333333
 .333333333333333
 .333333333333333
 4.33333333333333
 4.33333333333333
 4.33333333333333
 631
 606.666666666667
 61.3333333333333
 32
 29.3333333333333
 522.666666666667
 522.666666666667
 1.66666666666667
 1.66666666666667
 21
 20.3333333333333
 .666666666666667
 24.3333333333333
 .333333333333333
 .333333333333333
 24
 24
 1.33333333333333
 1.33333333333333
 1.33333333333333
 1.33333333333333
 57.3333333333333
 57.3333333333333
 57.3333333333333
 57.3333333333333
 .666666666666667
 .666666666666667
 .666666666666667
 .666666666666667
 1369.33333333333
 600
 7.66666666666667
 7.66666666666667
 6.66666666666667
 .333333333333333
 .333333333333333
 .333333333333333
 145.666666666667
 61
 54.6666666666667
 .333333333333333
 5.33333333333333
 .666666666666667
 81
 43.3333333333333
 9.33333333333333
 1.66666666666667
 4.66666666666667
 1
 2.33333333333333
 .333333333333333
 2.33333333333333
 1.33333333333333
 2.66666666666667
 4.66666666666667
 3
 2.66666666666667
 1.66666666666667
 2
 2
 1.66666666666667
 1
 .666666666666667
 15.6666666666667
 15.6666666666667
 15.6666666666667
 355.666666666667
 355.666666666667
 355.666666666667
 15.3333333333333
 15.3333333333333
 15.3333333333333
 2.66666666666667
 2.33333333333333
 2.33333333333333
 .333333333333333
 .333333333333333
 33.3333333333333
 33.3333333333333
 29
 4.33333333333333
 4.33333333333333
 4.33333333333333
 4.33333333333333
 16.6666666666667
 16.6666666666667
 16.6666666666667
 .333333333333333
 .333333333333333
 .333333333333333
 .666666666666667
 .666666666666667
 .666666666666667
 2
 2
 2
 267
 94.6666666666667
 5.33333333333333
 4.66666666666667
 .666666666666667
 16.3333333333333
 16.3333333333333
 18.3333333333333
 15.3333333333333
 .333333333333333
 1.66666666666667
 1
 2
 1.66666666666667
 .333333333333333
 4.33333333333333
 3.33333333333333
 1
 1
 .333333333333333
 .666666666666667
 .666666666666667
 .333333333333333
 .333333333333333
 12
 12
 34.6666666666667
 34.6666666666667
 1.33333333333333
 .666666666666667
 .666666666666667
 .666666666666667
 .666666666666667
 145.666666666667
 122.333333333333
 121
 .333333333333333
 1
 5.33333333333333
 .333333333333333
 5
 18
 18
 12.3333333333333
 11.3333333333333
 3.33333333333333
 3
 1
 3.33333333333333
 .666666666666667
 1
 1
 .666666666666667
 .333333333333333
 .333333333333333
 .333333333333333
 .333333333333333
 .666666666666667
 .666666666666667
 .666666666666667
 4.33333333333333
 4.33333333333333
 4.33333333333333
 6.66666666666667
 6.66666666666667
 6.66666666666667
 .666666666666667
 .666666666666667
 .333333333333333
 .333333333333333
 270.333333333333
 171
 91
 38.3333333333333
 52.6666666666667
 80
 5
 60.6666666666667
 1.33333333333333
 2.33333333333333
 3.66666666666667
 7
 1
 .333333333333333
 .333333333333333
 .666666666666667
 .666666666666667
 1.33333333333333
 1.33333333333333
 1.33333333333333
 2.66666666666667
 2.66666666666667
 2.66666666666667
 .333333333333333
 .333333333333333
 .333333333333333
 4.33333333333333
 4.33333333333333
 4.33333333333333
 3.66666666666667
 3.66666666666667
 3.66666666666667
 86
 86
 85.3333333333333
 .666666666666667
 232
 201.333333333333
 157.666666666667
 157.666666666667
 13
 13
 16
 16
 2.33333333333333
 1.66666666666667
 .666666666666667
 12
 4.33333333333333
 7.66666666666667
 .333333333333333
 .333333333333333
 18.3333333333333
 18.3333333333333
 17.6666666666667
 .666666666666667
 1.33333333333333
 1.33333333333333
 .333333333333333
 1
 .666666666666667
 .666666666666667
 .666666666666667
 1
 .333333333333333
 .333333333333333
 .666666666666667
 .666666666666667
 .333333333333333
 .333333333333333
 .333333333333333
 .666666666666667
 .666666666666667
 .666666666666667
 4
 4
 4
 4.33333333333333
 4.33333333333333
 4.33333333333333
 64.6666666666667
 64.3333333333333
 61
 59.3333333333333
 59.3333333333333
 1.66666666666667
 .666666666666667
 1
 3
 3
 2
 1
 .333333333333333
 .333333333333333
 .333333333333333
 .333333333333333
 .333333333333333
 .333333333333333
 .333333333333333
 57.6666666666667
 55
 54
 25
 20.3333333333333
 .666666666666667
 .666666666666667
 3.33333333333333
 .333333333333333
 .333333333333333
 18
 6
 10
 2
 2.66666666666667
 2.33333333333333
 .333333333333333
 5.33333333333333
 .666666666666667
 1.33333333333333
 1.66666666666667
 1
 .666666666666667
 1
 1
 1.66666666666667
 1.66666666666667
 1
 1
 1
 2.66666666666667
 2.66666666666667
 .333333333333333
 .333333333333333
 .333333333333333
 .333333333333333
 1.66666666666667
 1.66666666666667
 .333333333333333
 .333333333333333
 516
 335.666666666667
 250
 250
 250
 1
 1
 1
 6.66666666666667
 6.66666666666667
 6.66666666666667
 12.3333333333333
 12.3333333333333
 12.3333333333333
 62
 1.33333333333333
 1.33333333333333
 55.6666666666667
 55.6666666666667
 4.33333333333333
 4.33333333333333
 .666666666666667
 .666666666666667
 3.66666666666667
 3.66666666666667
 3.66666666666667
 3.66666666666667
 3.66666666666667
 3.66666666666667
 3.66666666666667
 92.3333333333333
 92.3333333333333
 92.3333333333333
 92.3333333333333
 38.6666666666667
 38.6666666666667
 38.6666666666667
 38.6666666666667
 29.6666666666667
 29.6666666666667
 29.6666666666667
 29.6666666666667
 16
 16
 16
 16
 1.66666666666667
 1.66666666666667
 1.66666666666667
 1.66666666666667
 1.66666666666667
 157.333333333333
 18.6666666666667
 18.6666666666667
 18.6666666666667
 18.6666666666667
 65
 31.6666666666667
 31.6666666666667
 31.6666666666667
 33.3333333333333
 33.3333333333333
 33.3333333333333
 3
 3
 3
 3
 10.3333333333333
 2.66666666666667
 2.33333333333333
 2.33333333333333
 .333333333333333
 .333333333333333
 6.66666666666667
 .666666666666667
 .666666666666667
 6
 6
 1
 1
 1
 4
 .666666666666667
 .666666666666667
 .666666666666667
 .333333333333333
 .333333333333333
 .333333333333333
 1.33333333333333
 1.33333333333333
 1.33333333333333
 1.66666666666667
 1.66666666666667
 1.66666666666667
 12
 12
 12
 12
 .333333333333333
 .333333333333333
 .333333333333333
 .333333333333333
 42.3333333333333
 15
 13.3333333333333
 13.3333333333333
 1.66666666666667
 1.66666666666667
 27.3333333333333
 27.3333333333333
 27.3333333333333
 .333333333333333
 .333333333333333
 .333333333333333
 .333333333333333
 1.33333333333333
 1.33333333333333
 1.33333333333333
 1.33333333333333
 7
 5
 5
 5
 5
 .666666666666667
 .666666666666667
 .666666666666667
 .666666666666667
 1
 1
 .333333333333333
 .333333333333333
 .666666666666667
 .666666666666667
 .333333333333333
 .333333333333333
 .333333333333333
 .333333333333333
 105
 105
 105
 47
 47
 58
 17.3333333333333
 40.6666666666667
 17.6666666666667
 12
 12
 12
 12
 .333333333333333
 .333333333333333
 .333333333333333
 .333333333333333
 1
 1
 1
 1
 4.33333333333333
 4.33333333333333
 4.33333333333333
 2
 2.33333333333333
 768.666666666667
 26.3333333333333
 23
 .333333333333333
 .333333333333333
 22.3333333333333
 22.3333333333333
 .333333333333333
 .333333333333333
 3.33333333333333
 3.33333333333333
 3.33333333333333
 260
 259.333333333333
 240.666666666667
 240.666666666667
 18.6666666666667
 18.6666666666667
 .666666666666667
 .666666666666667
 .666666666666667
 472.333333333333
 472.333333333333
 386.333333333333
 386.333333333333
 82
 82
 .333333333333333
 .333333333333333
 2.66666666666667
 2.66666666666667
 1
 1
 1.66666666666667
 1.66666666666667
 1.66666666666667
 1.66666666666667
 .333333333333333
 .333333333333333
 .333333333333333
 .333333333333333
 .333333333333333
 .333333333333333
 .333333333333333
 .333333333333333
 .333333333333333
 .333333333333333
 .333333333333333
 .333333333333333
 7.33333333333333
 7.33333333333333
 7.33333333333333
 1
 6.33333333333333
 4
 .666666666666667
 .666666666666667
 .666666666666667
 .333333333333333
 .333333333333333
 1.33333333333333
 1.33333333333333
 1.33333333333333
 1.33333333333333
 2
 2
 2
 2
 42.6666666666667
 19.3333333333333
 19.3333333333333
 18.6666666666667
 1.66666666666667
 4
 1.66666666666667
 11.3333333333333
 .666666666666667
 .666666666666667
 15.3333333333333
 15.3333333333333
 15.3333333333333
 8.33333333333333
 1
 6
 5
 5
 5
 5
 1
 1
 1
 1
 2
 2
 2
 2
 22.6666666666667
 22.6666666666667
 22.6666666666667
 22.6666666666667
 22.6666666666667
 1.66666666666667
 1.66666666666667
 1.66666666666667
 1.66666666666667
 1.66666666666667
 .333333333333333
 .333333333333333
 .333333333333333
 .333333333333333
 .333333333333333
 .333333333333333
 .333333333333333
 .333333333333333
 .333333333333333
 .333333333333333
 1.66666666666667
 1.66666666666667
 1.66666666666667
 1.66666666666667
 1.66666666666667
 202
 202
 202
 202
 202
 172.666666666667
 29.3333333333333
