# Supplementary material for: Response of Tomato Rhizosphere Bacteria to Root-Knot Nematodes, Fenamiphos and Sampling Time Shows Differential Effects on Low Level Taxa
Source: Front Microbiol. 2020 Mar 20;11:390. doi: 10.3389/fmicb.2020.00390 (PMC7100632; doi:10.3389/fmicb.2020.00390)
Supplement: FIGURE S2 — Interactive ring-charts (html format) produced with Krona, showing the mean taxonomic repartitions and relative abundance of taxa resulting from the RNAseq analyses, by treatment and sampling times. For treatments codes see legend of Supplementary Figure S1. Files constructed using the mean of three replications, except CON at time T0 (prior to transplants), and FEN-RKN at T2 (6 months), with two replicates each. Unclassified taxa were retained in the analyses. [file Presentation_2.zip › FEN T1 mean.html]

Javascript must be enabled to view this page.

magnitude
 3159.66666666667
 2996
 36.3333333333333
 36
 33.3333333333333
 13.3333333333333
 7
 1
 5.33333333333333
 12.3333333333333
 12.3333333333333
 5.33333333333333
 .333333333333333
 2.33333333333333
 .333333333333333
 .666666666666667
 1.66666666666667
 .333333333333333
 .333333333333333
 1.33333333333333
 1.33333333333333
 .666666666666667
 .666666666666667
 2.33333333333333
 2.33333333333333
 2.33333333333333
 .333333333333333
 .333333333333333
 .333333333333333
 .333333333333333
 .333333333333333
 .333333333333333
 .333333333333333
 166.666666666667
 166.666666666667
 166.666666666667
 73
 40.6666666666667
 32.3333333333333
 93.3333333333333
 93.3333333333333
 .333333333333333
 .333333333333333
 717.333333333333
 400.666666666667
 400.666666666667
 43.3333333333333
 43.3333333333333
 120.666666666667
 120.666666666667
 4.33333333333333
 3.66666666666667
 .666666666666667
 10.6666666666667
 8.33333333333333
 1.66666666666667
 .666666666666667
 80
 55
 22.3333333333333
 1
 1.66666666666667
 15.3333333333333
 11.6666666666667
 3.33333333333333
 .333333333333333
 59.3333333333333
 48.6666666666667
 3.33333333333333
 1
 6.33333333333333
 3.66666666666667
 1
 .666666666666667
 2
 24.6666666666667
 22
 2.66666666666667
 .333333333333333
 .333333333333333
 3.66666666666667
 2.66666666666667
 1
 .333333333333333
 .333333333333333
 3
 3
 2.66666666666667
 1.33333333333333
 1.33333333333333
 6
 6
 16
 1
 9.33333333333333
 5
 .666666666666667
 .333333333333333
 .333333333333333
 .666666666666667
 .666666666666667
 .666666666666667
 .666666666666667
 2.33333333333333
 2.33333333333333
 2.66666666666667
 2.66666666666667
 87
 87
 82
 82
 1.66666666666667
 1.66666666666667
 .666666666666667
 .666666666666667
 .333333333333333
 .333333333333333
 2.33333333333333
 2.33333333333333
 201.666666666667
 188.333333333333
 33.6666666666667
 21.6666666666667
 12
 143.666666666667
 143.666666666667
 11
 10.3333333333333
 .666666666666667
 13.3333333333333
 13.3333333333333
 13.3333333333333
 25
 25
 25
 24.3333333333333
 .666666666666667
 .333333333333333
 .333333333333333
 .333333333333333
 .333333333333333
 2.66666666666667
 2.66666666666667
 2.66666666666667
 2.66666666666667
 1131
 155.666666666667
 62
 1.33333333333333
 1
 .333333333333333
 54.6666666666667
 51.3333333333333
 1.33333333333333
 2
 6
 6
 71
 1.33333333333333
 1
 .333333333333333
 14.6666666666667
 14.6666666666667
 7.33333333333333
 6
 1
 .333333333333333
 44
 44
 .333333333333333
 .333333333333333
 2.33333333333333
 .666666666666667
 1.66666666666667
 1
 1
 1
 1
 1
 10.6666666666667
 9.66666666666667
 .333333333333333
 .666666666666667
 .333333333333333
 4.66666666666667
 3.66666666666667
 1
 1
 3
 3
 3
 .333333333333333
 .333333333333333
 .333333333333333
 7.66666666666667
 7.66666666666667
 7.66666666666667
 285.666666666667
 212.333333333333
 101
 68.6666666666667
 2.66666666666667
 1.66666666666667
 24
 2.33333333333333
 1
 .666666666666667
 111.333333333333
 28.6666666666667
 82.6666666666667
 11.6666666666667
 11.6666666666667
 9.33333333333333
 2.33333333333333
 .333333333333333
 .333333333333333
 .333333333333333
 3
 3
 3
 32
 32
 32
 25.3333333333333
 25.3333333333333
 25.3333333333333
 .333333333333333
 .333333333333333
 .333333333333333
 .666666666666667
 .666666666666667
 .666666666666667
 507.333333333333
 119
 74
 39.3333333333333
 2.33333333333333
 2.33333333333333
 2.66666666666667
 .333333333333333
 .666666666666667
 .666666666666667
 1.33333333333333
 7
 2.33333333333333
 .666666666666667
 1
 1
 2.66666666666667
 9.66666666666667
 38.3333333333333
 32.6666666666667
 5.66666666666667
 1.33333333333333
 1.33333333333333
 1.33333333333333
 1
 .333333333333333
 4
 4
 9.33333333333333
 9.33333333333333
 8
 .333333333333333
 .333333333333333
 .666666666666667
 152
 152
 152
 3.66666666666667
 3.66666666666667
 3.66666666666667
 3.66666666666667
 3.66666666666667
 3.66666666666667
 9
 9
 9
 196
 196
 188
 8
 2.66666666666667
 2.66666666666667
 2.66666666666667
 .333333333333333
 .333333333333333
 .333333333333333
 4.33333333333333
 4.33333333333333
 4.33333333333333
 2
 2
 2
 2.66666666666667
 2.66666666666667
 2.66666666666667
 2.66666666666667
 2.66666666666667
 2.66666666666667
 178.666666666667
 12.3333333333333
 12.3333333333333
 12
 .333333333333333
 1.33333333333333
 .333333333333333
 .333333333333333
 1
 1
 160.666666666667
 4.66666666666667
 4.66666666666667
 134
 134
 19.3333333333333
 2.33333333333333
 2.33333333333333
 14.6666666666667
 .333333333333333
 .333333333333333
 2.33333333333333
 2.33333333333333
 .666666666666667
 .666666666666667
 .666666666666667
 .666666666666667
 .666666666666667
 .666666666666667
 1.33333333333333
 1.33333333333333
 1.33333333333333
 1.66666666666667
 1.66666666666667
 1.66666666666667
 2.66666666666667
 2.66666666666667
 2.66666666666667
 2.66666666666667
 1
 1
 1
 1
 521.333333333333
 235
 235
 188
 188
 .333333333333333
 .333333333333333
 43.6666666666667
 43.6666666666667
 .666666666666667
 .666666666666667
 2.33333333333333
 2.33333333333333
 46.6666666666667
 45
 44.3333333333333
 44.3333333333333
 .333333333333333
 .333333333333333
 .333333333333333
 .333333333333333
 1.66666666666667
 1.66666666666667
 1.66666666666667
 230.666666666667
 230
 223.333333333333
 223.333333333333
 6.66666666666667
 6.66666666666667
 .666666666666667
 .666666666666667
 .666666666666667
 .333333333333333
 .333333333333333
 .333333333333333
 .333333333333333
 2
 2
 2
 2
 6.66666666666667
 6.66666666666667
 6.66666666666667
 2.66666666666667
 4
 9.33333333333333
 6
 6
 5.33333333333333
 .333333333333333
 1.66666666666667
 3
 .333333333333333
 .333333333333333
 .333333333333333
 .333333333333333
 .333333333333333
 1.33333333333333
 1.33333333333333
 1.33333333333333
 .333333333333333
 1
 .333333333333333
 .333333333333333
 .333333333333333
 .333333333333333
 1.66666666666667
 1.66666666666667
 1.66666666666667
 1.66666666666667
 157.333333333333
 93.6666666666667
 70.3333333333333
 70.3333333333333
 70.3333333333333
 16.3333333333333
 14.3333333333333
 14.3333333333333
 1.33333333333333
 1.33333333333333
 .666666666666667
 .666666666666667
 2.33333333333333
 2.33333333333333
 2.33333333333333
 2
 2
 2
 2.66666666666667
 2.66666666666667
 2.66666666666667
 2.33333333333333
 2.33333333333333
 2.33333333333333
 2.33333333333333
 9
 9
 9
 9
 27.3333333333333
 27.3333333333333
 27.3333333333333
 27.3333333333333
 5
 5
 5
 5
 20
 20
 20
 20
 2.66666666666667
 .333333333333333
 .333333333333333
 .333333333333333
 .333333333333333
 .333333333333333
 .333333333333333
 .333333333333333
 .333333333333333
 .666666666666667
 .666666666666667
 .666666666666667
 .666666666666667
 1.33333333333333
 1.33333333333333
 1.33333333333333
 1.33333333333333
 155.666666666667
 20
 20
 20
 20
 27
 6
 .666666666666667
 .666666666666667
 5
 5
 .333333333333333
 .333333333333333
 18.3333333333333
 1.33333333333333
 1.33333333333333
 17
 17
 2.66666666666667
 2.66666666666667
 2.66666666666667
 20
 9
 9
 9
 11
 9.66666666666667
 9.66666666666667
 1.33333333333333
 1.33333333333333
 2
 .333333333333333
 .333333333333333
 .333333333333333
 1.33333333333333
 1.33333333333333
 1.33333333333333
 .333333333333333
 .333333333333333
 .333333333333333
 14.3333333333333
 14.3333333333333
 14.3333333333333
 14.3333333333333
 69.3333333333333
 42.3333333333333
 42.3333333333333
 42.3333333333333
 27
 27
 27
 .333333333333333
 .333333333333333
 .333333333333333
 .333333333333333
 .666666666666667
 .666666666666667
 .666666666666667
 .666666666666667
 2
 2
 2
 2
 55.6666666666667
 .333333333333333
 .333333333333333
 .333333333333333
 .333333333333333
 53.3333333333333
 50.6666666666667
 50.3333333333333
 50.3333333333333
 .333333333333333
 .333333333333333
 .333333333333333
 .333333333333333
 .333333333333333
 2.33333333333333
 2.33333333333333
 1
 1.33333333333333
 1.66666666666667
 1.66666666666667
 1.66666666666667
 1.66666666666667
 .333333333333333
 .333333333333333
 .333333333333333
 .333333333333333
 22
 17.6666666666667
 17.6666666666667
 17.6666666666667
 17.6666666666667
 3
 3
 .333333333333333
 .333333333333333
 2.66666666666667
 2.66666666666667
 1.33333333333333
 1.33333333333333
 1.33333333333333
 1.33333333333333
 17.3333333333333
 .333333333333333
 .333333333333333
 .333333333333333
 .333333333333333
 1
 1
 1
 1
 16
 16
 16
 16
 .333333333333333
 .333333333333333
 .333333333333333
 .333333333333333
 .333333333333333
 1
 1
 1
 1
 1
 2
 2
 2
 2
 2
 163.666666666667
 163.666666666667
 163.666666666667
 163.666666666667
 163.666666666667
 141
 22.6666666666667
