# Supplementary material for: Response of Tomato Rhizosphere Bacteria to Root-Knot Nematodes, Fenamiphos and Sampling Time Shows Differential Effects on Low Level Taxa
Source: Front Microbiol. 2020 Mar 20;11:390. doi: 10.3389/fmicb.2020.00390 (PMC7100632; doi:10.3389/fmicb.2020.00390)
Supplement: FIGURE S2 — Interactive ring-charts (html format) produced with Krona, showing the mean taxonomic repartitions and relative abundance of taxa resulting from the RNAseq analyses, by treatment and sampling times. For treatments codes see legend of Supplementary Figure S1. Files constructed using the mean of three replications, except CON at time T0 (prior to transplants), and FEN-RKN at T2 (6 months), with two replicates each. Unclassified taxa were retained in the analyses. [file Presentation_2.zip › FEN T2 mean.html]

Javascript must be enabled to view this page.

magnitude
 4999.66666666667
 4999
 3395
 544.666666666667
 30.3333333333333
 30.3333333333333
 26
 .333333333333333
 .333333333333333
 .333333333333333
 1
 2.33333333333333
 4
 4
 4
 198
 44.3333333333333
 40.3333333333333
 1
 3
 144
 79.3333333333333
 1
 28.6666666666667
 5.33333333333333
 12.6666666666667
 .666666666666667
 .333333333333333
 2.33333333333333
 1.33333333333333
 .666666666666667
 4
 1.66666666666667
 1.33333333333333
 1.66666666666667
 3
 .333333333333333
 .333333333333333
 2.33333333333333
 2.33333333333333
 7
 1.33333333333333
 5.66666666666667
 18
 18
 2.66666666666667
 15.3333333333333
 256.333333333333
 256.333333333333
 256.333333333333
 .333333333333333
 .333333333333333
 .333333333333333
 10.6666666666667
 10.6666666666667
 10.6666666666667
 .333333333333333
 .333333333333333
 .333333333333333
 .333333333333333
 .333333333333333
 .333333333333333
 13
 13
 10.6666666666667
 2.33333333333333
 .333333333333333
 .333333333333333
 .333333333333333
 9.66666666666667
 9.66666666666667
 9.66666666666667
 3.33333333333333
 3.33333333333333
 3.33333333333333
 248.333333333333
 140.333333333333
 24.6666666666667
 23.3333333333333
 .333333333333333
 .666666666666667
 .333333333333333
 44
 36.6666666666667
 6.66666666666667
 .333333333333333
 .333333333333333
 20
 20
 23.6666666666667
 23.6666666666667
 16.3333333333333
 16.3333333333333
 4.66666666666667
 2.33333333333333
 .333333333333333
 1.33333333333333
 .666666666666667
 2.66666666666667
 .333333333333333
 2.33333333333333
 .333333333333333
 .333333333333333
 4
 .333333333333333
 3.66666666666667
 15
 13.3333333333333
 3
 3
 2
 .333333333333333
 4.33333333333333
 .666666666666667
 1.66666666666667
 1.66666666666667
 60
 59.3333333333333
 58.6666666666667
 .666666666666667
 .666666666666667
 .666666666666667
 14
 12.6666666666667
 12.6666666666667
 .666666666666667
 .666666666666667
 .666666666666667
 .666666666666667
 4.66666666666667
 4.66666666666667
 1
 2.33333333333333
 1.33333333333333
 1.33333333333333
 1.33333333333333
 .333333333333333
 1
 2
 2
 2
 9.33333333333333
 9.33333333333333
 9.33333333333333
 1.66666666666667
 1.66666666666667
 1.66666666666667
 171
 150.333333333333
 132.666666666667
 132.666666666667
 10.6666666666667
 10.6666666666667
 3.33333333333333
 3.33333333333333
 3.33333333333333
 2.33333333333333
 1
 .333333333333333
 .333333333333333
 9
 9
 9
 .333333333333333
 .333333333333333
 .333333333333333
 5.66666666666667
 5
 5
 .666666666666667
 .666666666666667
 2
 2
 2
 3.66666666666667
 3.66666666666667
 3.66666666666667
 2413
 .666666666666667
 .333333333333333
 .333333333333333
 .333333333333333
 .333333333333333
 225
 156.666666666667
 118.666666666667
 .666666666666667
 3
 6.33333333333333
 1
 1.33333333333333
 25.6666666666667
 68.3333333333333
 40
 28
 .333333333333333
 13
 9.66666666666667
 .333333333333333
 1.66666666666667
 7.66666666666667
 3.33333333333333
 1
 2.33333333333333
 2137.66666666667
 2137.66666666667
 2137.33333333333
 .333333333333333
 30.3333333333333
 30.3333333333333
 28.3333333333333
 2
 1
 .333333333333333
 .333333333333333
 .666666666666667
 .666666666666667
 4.66666666666667
 4.66666666666667
 4.66666666666667
 .333333333333333
 .333333333333333
 .333333333333333
 .333333333333333
 .333333333333333
 .333333333333333
 18
 18
 18
 3.66666666666667
 14.3333333333333
 106.666666666667
 93.3333333333333
 93.3333333333333
 84
 84
 6.33333333333333
 6.33333333333333
 .666666666666667
 .666666666666667
 1.66666666666667
 1.66666666666667
 .666666666666667
 .666666666666667
 1.33333333333333
 1
 .333333333333333
 .333333333333333
 .666666666666667
 .666666666666667
 .333333333333333
 .333333333333333
 .333333333333333
 .333333333333333
 .333333333333333
 .333333333333333
 .333333333333333
 8.66666666666667
 8.66666666666667
 4
 4
 4.66666666666667
 4.66666666666667
 .333333333333333
 .333333333333333
 .333333333333333
 .333333333333333
 .666666666666667
 .666666666666667
 .666666666666667
 .666666666666667
 2
 2
 2
 2
 831.333333333333
 197
 197
 186.666666666667
 186.666666666667
 3
 3
 1.33333333333333
 1.33333333333333
 2.33333333333333
 2.33333333333333
 3
 3
 .666666666666667
 .666666666666667
 502
 501
 3.66666666666667
 3.33333333333333
 .333333333333333
 19
 19
 5.66666666666667
 2.33333333333333
 2
 1.33333333333333
 53
 7
 39
 5
 2
 4.33333333333333
 2
 2
 .333333333333333
 14.6666666666667
 12.6666666666667
 2
 67
 50
 1.66666666666667
 15.3333333333333
 13
 1.33333333333333
 8.66666666666667
 2
 1
 85.6666666666667
 80
 5.66666666666667
 1
 .333333333333333
 .666666666666667
 6
 .333333333333333
 3.66666666666667
 .333333333333333
 .666666666666667
 1
 20.6666666666667
 2.33333333333333
 18.3333333333333
 84
 84
 1
 1
 100.666666666667
 9.33333333333333
 .333333333333333
 .666666666666667
 .666666666666667
 15.6666666666667
 74
 1.33333333333333
 .333333333333333
 1
 3.33333333333333
 3.33333333333333
 .333333333333333
 .333333333333333
 .666666666666667
 .333333333333333
 .333333333333333
 8
 8
 .666666666666667
 .666666666666667
 3
 3
 1
 1
 3.33333333333333
 3.33333333333333
 1
 1
 1
 115.333333333333
 109
 23
 11.3333333333333
 11.6666666666667
 84.6666666666667
 84.6666666666667
 1
 .666666666666667
 .333333333333333
 .333333333333333
 .333333333333333
 6.33333333333333
 6
 6
 .333333333333333
 .333333333333333
 13
 13
 13
 13
 3.66666666666667
 3.66666666666667
 3.66666666666667
 3.66666666666667
 .333333333333333
 .333333333333333
 .333333333333333
 .333333333333333
 73.6666666666667
 10.3333333333333
 10.3333333333333
 4
 4
 .333333333333333
 .333333333333333
 2
 2
 4
 1
 1
 2
 15.3333333333333
 15.3333333333333
 15.3333333333333
 3.66666666666667
 .666666666666667
 11
 41.6666666666667
 41.6666666666667
 38.3333333333333
 38.3333333333333
 1
 1
 1
 1
 1.33333333333333
 1.33333333333333
 1
 1
 1
 1
 5.33333333333333
 5.33333333333333
 .333333333333333
 .333333333333333
 2.33333333333333
 2.33333333333333
 2.66666666666667
 2.66666666666667
 .333333333333333
 .333333333333333
 .333333333333333
 .333333333333333
 .333333333333333
 196
 61.3333333333334
 56.6666666666667
 32.3333333333333
 22.6666666666667
 3.33333333333333
 1
 .333333333333333
 5
 3.33333333333333
 3.33333333333333
 8
 5.33333333333333
 .333333333333333
 .666666666666667
 1.66666666666667
 4.33333333333333
 2.66666666666667
 1.66666666666667
 8.33333333333333
 8.33333333333333
 .333333333333333
 .333333333333333
 4.66666666666667
 4.66666666666667
 4.66666666666667
 129.333333333333
 129.333333333333
 36.3333333333333
 24.3333333333333
 12
 46.3333333333333
 15.6666666666667
 9.66666666666667
 1
 20
 35.6666666666667
 35.6666666666667
 .333333333333333
 .333333333333333
 8.33333333333333
 3
 5.33333333333333
 1
 1
 1.33333333333333
 1.33333333333333
 5.33333333333333
 5.33333333333333
 5.33333333333333
 5.33333333333333
 13
 1.66666666666667
 1.66666666666667
 1.66666666666667
 1.66666666666667
 3.33333333333333
 1.33333333333333
 1.33333333333333
 1.33333333333333
 2
 2
 2
 4
 4
 4
 4
 .333333333333333
 .333333333333333
 .333333333333333
 .333333333333333
 .333333333333333
 .333333333333333
 .333333333333333
 .333333333333333
 .333333333333333
 .333333333333333
 .333333333333333
 .333333333333333
 2
 1.33333333333333
 .333333333333333
 .333333333333333
 1
 1
 .666666666666667
 .666666666666667
 .666666666666667
 1
 1
 1
 1
 192
 79
 17.6666666666667
 14
 14
 3.66666666666667
 3.66666666666667
 52.6666666666667
 52.6666666666667
 52.6666666666667
 6
 6
 6
 .666666666666667
 .666666666666667
 .666666666666667
 2
 2
 2
 8
 8
 8
 8
 5
 5
 5
 5
 16.3333333333333
 16.3333333333333
 16.3333333333333
 16.3333333333333
 69.3333333333333
 69.3333333333333
 69.3333333333333
 69.3333333333333
 14.3333333333333
 14.3333333333333
 14.3333333333333
 14.3333333333333
 1.33333333333333
 1.33333333333333
 1.33333333333333
 1.33333333333333
 1.33333333333333
 6
 3.33333333333333
 3.33333333333333
 3.33333333333333
 3.33333333333333
 2.66666666666667
 2.66666666666667
 2.66666666666667
 2.66666666666667
 34
 34
 34
 3.66666666666667
 3.66666666666667
 30.3333333333333
 22.6666666666667
 7.66666666666667
 2.66666666666667
 2
 .666666666666667
 .666666666666667
 .333333333333333
 .333333333333333
 1.33333333333333
 1.33333333333333
 1.33333333333333
 .333333333333333
 .333333333333333
 .333333333333333
 .333333333333333
 .333333333333333
 .333333333333333
 .333333333333333
 .333333333333333
 5.33333333333333
 5.33333333333333
 .666666666666667
 .666666666666667
 .666666666666667
 4.66666666666667
 4.66666666666667
 4.66666666666667
 3.33333333333333
 3.33333333333333
 3.33333333333333
 3.33333333333333
 3.33333333333333
 138.333333333333
 138.333333333333
 138.333333333333
 138.333333333333
 138.333333333333
 .666666666666667
 .666666666666667
 .666666666666667
 .666666666666667
 .666666666666667
 .666666666666667
