# Supplementary material for: Response of Tomato Rhizosphere Bacteria to Root-Knot Nematodes, Fenamiphos and Sampling Time Shows Differential Effects on Low Level Taxa
Source: Front Microbiol. 2020 Mar 20;11:390. doi: 10.3389/fmicb.2020.00390 (PMC7100632; doi:10.3389/fmicb.2020.00390)
Supplement: FIGURE S2 — Interactive ring-charts (html format) produced with Krona, showing the mean taxonomic repartitions and relative abundance of taxa resulting from the RNAseq analyses, by treatment and sampling times. For treatments codes see legend of Supplementary Figure S1. Files constructed using the mean of three replications, except CON at time T0 (prior to transplants), and FEN-RKN at T2 (6 months), with two replicates each. Unclassified taxa were retained in the analyses. [file Presentation_2.zip › FEN RKN T1 mean.html]

Javascript must be enabled to view this page.

magnitude
 3735.66666666667
 3643.33333333334
 812.333333333333
 376
 376
 316
 316
 40.3333333333333
 40.3333333333333
 .333333333333333
 .333333333333333
 .666666666666667
 .666666666666667
 18.6666666666667
 18.6666666666667
 73
 72.3333333333333
 71.3333333333333
 71.3333333333333
 1
 1
 .666666666666667
 .666666666666667
 .666666666666667
 359.333333333333
 .666666666666667
 .666666666666667
 .666666666666667
 358.333333333333
 354.666666666667
 354.666666666667
 3.66666666666667
 3.66666666666667
 .333333333333333
 .333333333333333
 .333333333333333
 2.33333333333333
 2.33333333333333
 2.33333333333333
 1.33333333333333
 1
 .333333333333333
 .333333333333333
 .333333333333333
 .333333333333333
 .333333333333333
 .333333333333333
 .333333333333333
 .333333333333333
 .333333333333333
 .333333333333333
 .333333333333333
 .333333333333333
 .666666666666667
 .666666666666667
 .666666666666667
 .666666666666667
 1237.33333333333
 374
 90.6666666666667
 90.6666666666667
 83.6666666666667
 7
 4.66666666666667
 4.66666666666667
 4.33333333333333
 .333333333333333
 84
 54.3333333333333
 8
 29.6666666666667
 1.66666666666667
 1.66666666666667
 .666666666666667
 .333333333333333
 2.66666666666667
 2.66666666666667
 3.66666666666667
 3.33333333333333
 25.3333333333333
 23.3333333333333
 .333333333333333
 1.66666666666667
 .666666666666667
 .666666666666667
 3
 3
 .666666666666667
 .666666666666667
 2
 2
 2
 8.33333333333333
 8.33333333333333
 8.33333333333333
 163.666666666667
 163.666666666667
 163.666666666667
 .666666666666667
 .666666666666667
 .666666666666667
 .333333333333333
 .333333333333333
 .333333333333333
 1.66666666666667
 1.66666666666667
 1.66666666666667
 9.33333333333333
 9.33333333333333
 9.33333333333333
 1.66666666666667
 1.66666666666667
 1.66666666666667
 1.66666666666667
 1.66666666666667
 1.66666666666667
 5.33333333333333
 5.33333333333333
 5.33333333333333
 216.666666666667
 166
 45.6666666666667
 36
 1
 2.66666666666667
 3.66666666666667
 .333333333333333
 2
 120.333333333333
 32
 88.3333333333333
 22.6666666666667
 22.6666666666667
 .333333333333333
 22.3333333333333
 14.3333333333333
 14
 .333333333333333
 13.6666666666667
 .333333333333333
 .333333333333333
 .666666666666667
 .666666666666667
 .333333333333333
 .333333333333333
 .666666666666667
 .333333333333333
 .333333333333333
 .333333333333333
 .333333333333333
 12
 12
 12
 .333333333333333
 .333333333333333
 .333333333333333
 401.666666666667
 247.666666666667
 2
 1.66666666666667
 .333333333333333
 242
 235.666666666667
 5.66666666666667
 .666666666666667
 3.66666666666667
 3.66666666666667
 2.33333333333333
 2.33333333333333
 2
 .333333333333333
 128.333333333333
 10
 5.66666666666667
 .333333333333333
 3.66666666666667
 .333333333333333
 9
 8
 .666666666666667
 .333333333333333
 13
 13
 2
 .666666666666667
 .333333333333333
 1
 15
 15
 77.3333333333333
 77.3333333333333
 .666666666666667
 .333333333333333
 .333333333333333
 1
 1
 .333333333333333
 .333333333333333
 7.33333333333333
 1
 .666666666666667
 .333333333333333
 6.33333333333333
 6.33333333333333
 1
 1
 1
 6
 5.66666666666667
 3
 .666666666666667
 2
 .333333333333333
 .333333333333333
 8.33333333333333
 8.33333333333333
 8.33333333333333
 .333333333333333
 .333333333333333
 .333333333333333
 .333333333333333
 .333333333333333
 .333333333333333
 245
 64
 64
 62.6666666666667
 1.33333333333333
 171.333333333333
 154.333333333333
 154.333333333333
 5.66666666666667
 5.66666666666667
 1
 1
 1
 .333333333333333
 .666666666666667
 9.33333333333333
 9.33333333333333
 .333333333333333
 .333333333333333
 .333333333333333
 1.66666666666667
 .333333333333333
 .333333333333333
 1.33333333333333
 1.33333333333333
 1.66666666666667
 1.66666666666667
 1
 .666666666666667
 .333333333333333
 .333333333333333
 .333333333333333
 1
 1
 1
 1.33333333333333
 1.33333333333333
 1.33333333333333
 3.33333333333333
 3.33333333333333
 3.33333333333333
 765.333333333333
 94.6666666666667
 94.6666666666667
 87.6666666666667
 87.6666666666667
 2.33333333333333
 2.33333333333333
 2.66666666666667
 2.66666666666667
 1.33333333333333
 1.33333333333333
 .333333333333333
 .333333333333333
 .333333333333333
 .333333333333333
 396
 395.333333333333
 3
 .333333333333333
 2.66666666666667
 115.333333333333
 95.6666666666667
 13.6666666666667
 1
 5
 .666666666666667
 .666666666666667
 8.33333333333333
 4.66666666666667
 .333333333333333
 2.33333333333333
 1
 17
 11.6666666666667
 .666666666666667
 4.66666666666667
 23
 15.3333333333333
 7.66666666666667
 4.66666666666667
 1.66666666666667
 3
 3.33333333333333
 2.33333333333333
 1
 42
 42
 1.66666666666667
 .333333333333333
 .333333333333333
 1
 139.666666666667
 .333333333333333
 101
 36
 1
 1.33333333333333
 1
 1
 1.33333333333333
 .333333333333333
 .333333333333333
 .666666666666667
 1.66666666666667
 .666666666666667
 1
 1.66666666666667
 1
 .666666666666667
 .333333333333333
 .333333333333333
 19.6666666666667
 5.33333333333333
 1
 .333333333333333
 13
 1
 .333333333333333
 .666666666666667
 .333333333333333
 .333333333333333
 4.66666666666667
 1.66666666666667
 3
 3
 3
 2
 .666666666666667
 1.33333333333333
 .666666666666667
 .666666666666667
 .666666666666667
 2.66666666666667
 2.66666666666667
 2.66666666666667
 2.66666666666667
 191.666666666667
 177.666666666667
 71
 38
 33
 7.66666666666667
 7.66666666666667
 98.6666666666667
 98.6666666666667
 .333333333333333
 .333333333333333
 14
 14
 14
 80
 80
 80
 78.3333333333333
 1.66666666666667
 .333333333333333
 .333333333333333
 .333333333333333
 .333333333333333
 77.3333333333333
 75.3333333333333
 71.3333333333333
 70.6666666666667
 70.6666666666667
 .666666666666667
 .333333333333333
 .333333333333333
 3.33333333333333
 3.33333333333333
 1.66666666666667
 1.66666666666667
 .666666666666667
 .666666666666667
 .666666666666667
 1.66666666666667
 1
 1
 1
 .666666666666667
 .666666666666667
 .666666666666667
 .333333333333333
 .333333333333333
 .333333333333333
 .333333333333333
 53.6666666666667
 52
 52
 17.6666666666667
 16
 1.33333333333333
 .333333333333333
 2
 2
 2.33333333333333
 2
 .333333333333333
 23.6666666666667
 6.66666666666667
 15.3333333333333
 1.66666666666667
 6.33333333333333
 1.33333333333333
 .333333333333333
 .333333333333333
 .666666666666667
 3.66666666666667
 1.66666666666667
 1.66666666666667
 .333333333333333
 .333333333333333
 .333333333333333
 .333333333333333
 1
 1
 129
 20.6666666666667
 20.6666666666667
 20.6666666666667
 20.6666666666667
 35.3333333333333
 .333333333333333
 .333333333333333
 .333333333333333
 2.66666666666667
 1.66666666666667
 1.66666666666667
 .333333333333333
 .333333333333333
 .666666666666667
 .666666666666667
 .666666666666667
 .666666666666667
 .666666666666667
 30.6666666666667
 30.6666666666667
 30.6666666666667
 1
 1
 1
 21.6666666666667
 21.6666666666667
 21.6666666666667
 21.6666666666667
 37.6666666666667
 37.6666666666667
 37.6666666666667
 37.6666666666667
 1.33333333333333
 1.33333333333333
 1.33333333333333
 1.33333333333333
 12.3333333333333
 12.3333333333333
 12.3333333333333
 12.3333333333333
 2
 .333333333333333
 .333333333333333
 .333333333333333
 .333333333333333
 1
 1
 1
 1
 .333333333333333
 .333333333333333
 .333333333333333
 .333333333333333
 .333333333333333
 .333333333333333
 .333333333333333
 .333333333333333
 22
 2
 2
 2
 1
 .333333333333333
 .666666666666667
 17
 17
 9.33333333333333
 5.66666666666667
 3
 .666666666666667
 5.33333333333333
 5.33333333333333
 1
 1
 1.33333333333333
 1.33333333333333
 1.33333333333333
 1.33333333333333
 1.33333333333333
 1.33333333333333
 1.66666666666667
 1.66666666666667
 .666666666666667
 .666666666666667
 1
 1
 295
 109
 63.3333333333333
 63.3333333333333
 63.3333333333333
 45.6666666666667
 45.6666666666667
 45.6666666666667
 6.66666666666667
 .333333333333333
 .333333333333333
 .333333333333333
 2.33333333333333
 2.33333333333333
 2.33333333333333
 2.33333333333333
 2.33333333333333
 2.33333333333333
 .666666666666667
 .666666666666667
 .666666666666667
 1
 1
 1
 62.3333333333333
 62.3333333333333
 62.3333333333333
 62.3333333333333
 10
 10
 10
 10
 36.6666666666667
 9.33333333333333
 9
 9
 .333333333333333
 .333333333333333
 23.3333333333333
 22.6666666666667
 22.6666666666667
 .666666666666667
 .666666666666667
 4
 4
 4
 30.3333333333333
 30.3333333333333
 30.3333333333333
 30.3333333333333
 .333333333333333
 .333333333333333
 .333333333333333
 .333333333333333
 33.6666666666667
 11.3333333333333
 11.3333333333333
 11.3333333333333
 22.3333333333333
 21.6666666666667
 21.6666666666667
 .666666666666667
 .666666666666667
 1.33333333333333
 1.33333333333333
 1.33333333333333
 1.33333333333333
 1.66666666666667
 1.66666666666667
 1.66666666666667
 1.66666666666667
 3
 3
 3
 3
 15
 12
 12
 .333333333333333
 .333333333333333
 11
 11
 .666666666666667
 .666666666666667
 2.66666666666667
 2.66666666666667
 .333333333333333
 .333333333333333
 2.33333333333333
 2.33333333333333
 .333333333333333
 .333333333333333
 .333333333333333
 .333333333333333
 64.3333333333333
 .666666666666667
 .666666666666667
 .666666666666667
 .666666666666667
 36
 36
 36
 36
 3
 .333333333333333
 .333333333333333
 .333333333333333
 2.66666666666667
 2.66666666666667
 2.66666666666667
 24.6666666666667
 24.6666666666667
 24.6666666666667
 24.6666666666667
 153.333333333333
 153.333333333333
 153.333333333333
 46
 15.3333333333333
 30.6666666666667
 107.333333333333
 107.333333333333
 1
 1
 1
 1
 1
 .333333333333333
 .333333333333333
 .333333333333333
 .333333333333333
 .333333333333333
 .333333333333333
 .333333333333333
 .333333333333333
 .333333333333333
 .333333333333333
 .333333333333333
 .333333333333333
 .333333333333333
 .333333333333333
 .333333333333333
 2.33333333333333
 2.33333333333333
 2.33333333333333
 2.33333333333333
 2.33333333333333
 .333333333333333
 .333333333333333
 .333333333333333
 .333333333333333
 .333333333333333
 .333333333333333
 .333333333333333
 .333333333333333
 .333333333333333
 .333333333333333
 1.66666666666667
 1.66666666666667
 1.66666666666667
 1.66666666666667
 1.66666666666667
 .333333333333333
 .333333333333333
 .333333333333333
 .333333333333333
 .333333333333333
 .333333333333333
 .333333333333333
 .333333333333333
 .333333333333333
 .333333333333333
 3
 3
 3
 3
 3
 6.33333333333333
 6.33333333333333
 6.33333333333333
 6.33333333333333
 6.33333333333333
 92.3333333333333
 92.3333333333333
 92.3333333333333
 92.3333333333333
 92.3333333333333
 81.6666666666667
 10.6666666666667
