# Supplementary material for: Response of Tomato Rhizosphere Bacteria to Root-Knot Nematodes, Fenamiphos and Sampling Time Shows Differential Effects on Low Level Taxa
Source: Front Microbiol. 2020 Mar 20;11:390. doi: 10.3389/fmicb.2020.00390 (PMC7100632; doi:10.3389/fmicb.2020.00390)
Supplement: FIGURE S2 — Interactive ring-charts (html format) produced with Krona, showing the mean taxonomic repartitions and relative abundance of taxa resulting from the RNAseq analyses, by treatment and sampling times. For treatments codes see legend of Supplementary Figure S1. Files constructed using the mean of three replications, except CON at time T0 (prior to transplants), and FEN-RKN at T2 (6 months), with two replicates each. Unclassified taxa were retained in the analyses. [file Presentation_2.zip › FEN RKN T2 mean.html]

Javascript must be enabled to view this page.

magnitude
 6441.00000000001
 6440.00000000001
 1482
 2
 2
 2
 2
 528
 526
 8.33333333333333
 .333333333333333
 8
 24.3333333333333
 .333333333333333
 13.3333333333333
 .666666666666667
 8.33333333333333
 1.66666666666667
 7.66666666666667
 3.66666666666667
 3
 1
 13
 4
 .333333333333333
 1.66666666666667
 5.66666666666667
 .333333333333333
 .333333333333333
 .666666666666667
 39.6666666666667
 25
 1.66666666666667
 2.33333333333333
 3
 7.66666666666667
 75.3333333333333
 57.3333333333333
 13.3333333333333
 .333333333333333
 .333333333333333
 1
 3
 163.333333333333
 163.333333333333
 .333333333333333
 .333333333333333
 60.6666666666667
 46.6666666666667
 14
 .666666666666667
 .666666666666667
 3
 .333333333333333
 1.66666666666667
 1
 42
 42
 24.6666666666667
 .666666666666667
 .666666666666667
 5.33333333333333
 16.6666666666667
 1.33333333333333
 7.66666666666667
 3
 2.66666666666667
 .333333333333333
 .333333333333333
 1.33333333333333
 13.3333333333333
 13.3333333333333
 .333333333333333
 .333333333333333
 2
 .333333333333333
 1.66666666666667
 .666666666666667
 .666666666666667
 2.33333333333333
 2.33333333333333
 .333333333333333
 .333333333333333
 34
 2
 8.66666666666667
 17.6666666666667
 5.66666666666667
 .333333333333333
 .333333333333333
 2
 2
 2
 2
 2
 539
 539
 531.333333333333
 531.333333333333
 1.33333333333333
 1.33333333333333
 2
 2
 2.66666666666667
 2.66666666666667
 1
 1
 .666666666666667
 .666666666666667
 283
 14.3333333333333
 14.3333333333333
 14.3333333333333
 268.666666666667
 197.333333333333
 197.333333333333
 57.6666666666667
 21.3333333333333
 36.3333333333333
 10.3333333333333
 10.3333333333333
 3.33333333333333
 3.33333333333333
 130
 130
 130
 129.666666666667
 .333333333333333
 3725.33333333333
 2371.33333333333
 23.3333333333333
 23
 22
 1
 .333333333333333
 .333333333333333
 1464.33333333333
 545.333333333333
 113.666666666667
 329
 4
 7.33333333333333
 .333333333333333
 10.3333333333333
 11
 9.33333333333333
 6.33333333333333
 9.33333333333333
 1.33333333333333
 2
 .333333333333333
 2
 3
 24.6666666666667
 8.66666666666667
 2.66666666666667
 902.333333333333
 705
 .333333333333333
 195.666666666667
 1.33333333333333
 10
 10
 6.66666666666667
 6.66666666666667
 3.33333333333333
 3.33333333333333
 3.33333333333333
 496.666666666667
 496.666666666667
 496.666666666667
 81.3333333333333
 81.3333333333333
 69
 5.66666666666667
 1.66666666666667
 .666666666666667
 .333333333333333
 .333333333333333
 .333333333333333
 1.66666666666667
 .333333333333333
 .666666666666667
 .666666666666667
 72.6666666666667
 72.6666666666667
 72.6666666666667
 1.66666666666667
 1.66666666666667
 1.66666666666667
 11
 11
 11
 25
 25
 25
 .666666666666667
 .666666666666667
 .666666666666667
 6
 6
 6
 .666666666666667
 .666666666666667
 .666666666666667
 181
 181
 138
 43
 3
 .333333333333333
 .333333333333333
 2.66666666666667
 2.66666666666667
 .666666666666667
 .666666666666667
 .666666666666667
 407.333333333333
 8.33333333333333
 8.33333333333333
 5
 1.66666666666667
 1.66666666666667
 141.333333333333
 12.6666666666667
 .666666666666667
 2.33333333333333
 9.33333333333333
 .333333333333333
 10.3333333333333
 7.66666666666667
 .333333333333333
 2.33333333333333
 28.3333333333333
 28.3333333333333
 1.66666666666667
 1.33333333333333
 .333333333333333
 47
 47
 9
 3
 5.66666666666667
 .333333333333333
 23.6666666666667
 23.6666666666667
 3
 .333333333333333
 2.66666666666667
 5.66666666666667
 2.66666666666667
 3
 199
 192
 191
 1
 6.66666666666667
 6.66666666666667
 .333333333333333
 .333333333333333
 23
 20.3333333333333
 .333333333333333
 8.66666666666667
 1.66666666666667
 2.33333333333333
 6.33333333333333
 1
 2.66666666666667
 2.66666666666667
 5.66666666666667
 .333333333333333
 .333333333333333
 5.33333333333333
 5.33333333333333
 2.33333333333333
 2.33333333333333
 2.33333333333333
 11
 11
 11
 5.33333333333333
 1.66666666666667
 .666666666666667
 1
 3.66666666666667
 3.66666666666667
 11.3333333333333
 11.3333333333333
 11.3333333333333
 566
 257.333333333333
 179
 147
 2.66666666666667
 1
 2.33333333333333
 2.66666666666667
 .333333333333333
 .333333333333333
 14.6666666666667
 3
 5
 78.3333333333333
 27
 51.3333333333333
 1.66666666666667
 1.66666666666667
 1.66666666666667
 35
 35
 30.3333333333333
 4.66666666666667
 .666666666666667
 .666666666666667
 .666666666666667
 9.33333333333333
 8
 4.33333333333333
 1.33333333333333
 .666666666666667
 1.66666666666667
 1.33333333333333
 1.33333333333333
 30
 30
 30
 229.333333333333
 4
 2.33333333333333
 1.66666666666667
 225.333333333333
 225.333333333333
 1
 1
 1
 1.66666666666667
 1.66666666666667
 1.66666666666667
 1.33333333333333
 1.33333333333333
 1.33333333333333
 1.33333333333333
 379.333333333333
 303.333333333333
 32.3333333333333
 32.3333333333333
 32.3333333333333
 32.3333333333333
 1.66666666666667
 1.66666666666667
 203
 203
 34
 15.3333333333333
 18.6666666666667
 4.66666666666667
 4.66666666666667
 2.66666666666667
 2
 .333333333333333
 .333333333333333
 .333333333333333
 48.3333333333333
 48
 48
 .333333333333333
 .333333333333333
 10
 10
 10
 3
 1.66666666666667
 1.33333333333333
 .333333333333333
 1.33333333333333
 1.33333333333333
 2.66666666666667
 1
 1
 1.66666666666667
 1.66666666666667
 1
 1
 1
 6
 6
 6
 475.666666666667
 70.6666666666667
 70.6666666666667
 70.6666666666667
 70.6666666666667
 306
 255.666666666667
 255.666666666667
 255.666666666667
 34.3333333333333
 31.3333333333333
 31.3333333333333
 1.66666666666667
 1.66666666666667
 1.33333333333333
 1.33333333333333
 5
 5
 5
 7.66666666666667
 7.66666666666667
 7.66666666666667
 .666666666666667
 .666666666666667
 .666666666666667
 2.66666666666667
 2.66666666666667
 2.66666666666667
 55.3333333333333
 55.3333333333333
 55.3333333333333
 55.3333333333333
 28
 28
 28
 28
 7
 7
 7
 7
 8.66666666666667
 8.66666666666667
 8.66666666666667
 8.66666666666667
 64.3333333333333
 63
 60
 11.3333333333333
 .333333333333333
 8.33333333333333
 2.33333333333333
 .333333333333333
 37.6666666666667
 30.3333333333333
 .333333333333333
 6.66666666666667
 .333333333333333
 .333333333333333
 .333333333333333
 3
 3
 5
 2.33333333333333
 .333333333333333
 1
 1.33333333333333
 2.66666666666667
 2.66666666666667
 3
 3
 3
 1.33333333333333
 1.33333333333333
 .333333333333333
 .333333333333333
 1
 1
 23
 .333333333333333
 .333333333333333
 .333333333333333
 .333333333333333
 .333333333333333
 .333333333333333
 .333333333333333
 .333333333333333
 12
 12
 12
 12
 .333333333333333
 .333333333333333
 .333333333333333
 .333333333333333
 2.33333333333333
 1.33333333333333
 .666666666666667
 .666666666666667
 .666666666666667
 .666666666666667
 1
 .333333333333333
 .333333333333333
 .666666666666667
 .666666666666667
 .666666666666667
 .666666666666667
 .666666666666667
 .666666666666667
 1
 1
 1
 1
 6
 2.33333333333333
 2.33333333333333
 2.33333333333333
 3.66666666666667
 3.66666666666667
 3.66666666666667
 40.6666666666667
 13
 13
 11.6666666666667
 7.33333333333333
 .333333333333333
 .333333333333333
 .666666666666667
 .666666666666667
 2.33333333333333
 1.33333333333333
 1.33333333333333
 12
 12
 6.66666666666667
 5.66666666666667
 1
 .333333333333333
 .333333333333333
 1.33333333333333
 1.33333333333333
 3.66666666666667
 3.66666666666667
 1.33333333333333
 1.33333333333333
 1.33333333333333
 1.33333333333333
 5
 5
 2
 .333333333333333
 1.66666666666667
 3
 2
 1
 9.33333333333333
 9.33333333333333
 9.33333333333333
 9.33333333333333
 7.66666666666667
 6.66666666666667
 6.66666666666667
 5.66666666666667
 1.33333333333333
 4.33333333333333
 1
 1
 .333333333333333
 .333333333333333
 .333333333333333
 .333333333333333
 .666666666666667
 .666666666666667
 .666666666666667
 .666666666666667
 385.333333333333
 371.333333333333
 371.333333333333
 71
 71
 18
 18
 280
 280
 .333333333333333
 .333333333333333
 1
 1
 1
 1
 5.66666666666667
 5.33333333333333
 5.33333333333333
 5.33333333333333
 .333333333333333
 .333333333333333
 .333333333333333
 .333333333333333
 .333333333333333
 .333333333333333
 .333333333333333
 .333333333333333
 .333333333333333
 .333333333333333
 .333333333333333
 1
 1
 1
 1
 .333333333333333
 .333333333333333
 .333333333333333
 .333333333333333
 6.33333333333333
 6.33333333333333
 6.33333333333333
 6.33333333333333
 4.66666666666667
 4.66666666666667
 4.66666666666667
 4.33333333333333
 4.33333333333333
 .333333333333333
 .333333333333333
 222
 222
 222
 215
 113.666666666667
 101.333333333333
 7
 7
 5.33333333333333
 5.33333333333333
 .333333333333333
 .333333333333333
 .333333333333333
 5
 5
 5
 2
 2
 2
 2
 2
 .333333333333333
 .333333333333333
 .333333333333333
 .333333333333333
 .333333333333333
 .666666666666667
 .666666666666667
 .666666666666667
 .666666666666667
 .666666666666667
 1
 1
 1
 1
 1
 1
 1
 1
 1
 1
 1
