# Supplementary material for: Response of Tomato Rhizosphere Bacteria to Root-Knot Nematodes, Fenamiphos and Sampling Time Shows Differential Effects on Low Level Taxa
Source: Front Microbiol. 2020 Mar 20;11:390. doi: 10.3389/fmicb.2020.00390 (PMC7100632; doi:10.3389/fmicb.2020.00390)
Supplement: FIGURE S2 — Interactive ring-charts (html format) produced with Krona, showing the mean taxonomic repartitions and relative abundance of taxa resulting from the RNAseq analyses, by treatment and sampling times. For treatments codes see legend of Supplementary Figure S1. Files constructed using the mean of three replications, except CON at time T0 (prior to transplants), and FEN-RKN at T2 (6 months), with two replicates each. Unclassified taxa were retained in the analyses. [file Presentation_2.zip › RKN T1 mean.html]

Javascript must be enabled to view this page.

magnitude
 3644
 3545
 905
 385
 64.3333333333333
 64.3333333333333
 60
 4.33333333333333
 76.6666666666667
 36.3333333333333
 16.3333333333333
 4
 1.66666666666667
 .333333333333333
 .333333333333333
 .333333333333333
 .333333333333333
 .666666666666667
 1
 1
 5
 2.66666666666667
 2.66666666666667
 .333333333333333
 .333333333333333
 38.3333333333333
 34.3333333333333
 3.66666666666667
 .333333333333333
 1.66666666666667
 1.66666666666667
 2.66666666666667
 2.66666666666667
 2.33333333333333
 .333333333333333
 4.33333333333333
 4.33333333333333
 4.33333333333333
 2
 2
 2
 6
 6
 6
 11.6666666666667
 11.6666666666667
 1.66666666666667
 10
 206
 206
 206
 8
 8
 8
 .666666666666667
 .666666666666667
 .666666666666667
 2.66666666666667
 2.66666666666667
 2.66666666666667
 176.333333333333
 .333333333333333
 .333333333333333
 .333333333333333
 18.6666666666667
 16.6666666666667
 .333333333333333
 13.6666666666667
 1.66666666666667
 .666666666666667
 .333333333333333
 2
 2
 69
 6
 4
 2
 3
 2.33333333333333
 .333333333333333
 .333333333333333
 6
 1.66666666666667
 4.33333333333333
 3.33333333333333
 3.33333333333333
 .333333333333333
 .333333333333333
 23
 23
 27.3333333333333
 27.3333333333333
 2
 2
 .333333333333333
 .333333333333333
 1.33333333333333
 78
 1.33333333333333
 1.33333333333333
 70.3333333333333
 68.6666666666667
 .333333333333333
 .333333333333333
 .333333333333333
 .666666666666667
 6.33333333333333
 6.33333333333333
 .666666666666667
 .666666666666667
 .666666666666667
 1
 1
 .333333333333333
 .666666666666667
 .333333333333333
 .333333333333333
 .333333333333333
 6.33333333333333
 6.33333333333333
 6.33333333333333
 .333333333333333
 .333333333333333
 .333333333333333
 .333333333333333
 80
 59.3333333333333
 .333333333333333
 .333333333333333
 50.6666666666667
 50.6666666666667
 6
 6
 1
 .666666666666667
 .333333333333333
 1.33333333333333
 1.33333333333333
 16
 16
 16
 .333333333333333
 .333333333333333
 .333333333333333
 .333333333333333
 .333333333333333
 .333333333333333
 .333333333333333
 .333333333333333
 .333333333333333
 2.33333333333333
 2.33333333333333
 1.33333333333333
 1
 .666666666666667
 .666666666666667
 .666666666666667
 .666666666666667
 .666666666666667
 .666666666666667
 263.333333333333
 21
 .333333333333333
 .333333333333333
 19.6666666666667
 19
 .666666666666667
 1
 1
 29
 29
 27.6666666666667
 1.33333333333333
 1.33333333333333
 1.33333333333333
 1.33333333333333
 184
 77.6666666666667
 42.3333333333333
 2.66666666666667
 5.33333333333333
 2
 24.6666666666667
 .666666666666667
 106.333333333333
 62.3333333333333
 44
 4.66666666666667
 4.66666666666667
 4
 .666666666666667
 23
 23
 23
 .333333333333333
 .333333333333333
 .333333333333333
 972
 455.333333333333
 455
 173.333333333333
 99
 5
 68.6666666666667
 .666666666666667
 7
 1.33333333333333
 4
 1
 .333333333333333
 .333333333333333
 38.3333333333333
 26
 12.3333333333333
 6
 4.66666666666667
 .333333333333333
 1
 5
 1.66666666666667
 3
 .333333333333333
 55
 43.6666666666667
 .333333333333333
 3.66666666666667
 2.33333333333333
 4
 1
 .666666666666667
 .333333333333333
 .333333333333333
 32.3333333333333
 32.3333333333333
 62
 62
 1.66666666666667
 .333333333333333
 1.33333333333333
 1
 1
 3.66666666666667
 .666666666666667
 3
 7.66666666666667
 7.66666666666667
 .333333333333333
 .333333333333333
 10.3333333333333
 .666666666666667
 7.33333333333333
 2.33333333333333
 5.66666666666667
 .333333333333333
 1
 4.33333333333333
 33.6666666666667
 18.6666666666667
 3
 9
 3
 3
 3
 1.33333333333333
 1.33333333333333
 .666666666666667
 .666666666666667
 .666666666666667
 .666666666666667
 3.66666666666667
 3.66666666666667
 2
 2
 .333333333333333
 .333333333333333
 .333333333333333
 346.333333333333
 315.333333333333
 5.66666666666667
 5
 .666666666666667
 257.666666666667
 257.666666666667
 52
 34
 18
 31
 31
 31
 140
 140
 131.333333333333
 131.333333333333
 2.33333333333333
 2.33333333333333
 4.33333333333333
 4.33333333333333
 1.33333333333333
 1.33333333333333
 .666666666666667
 .666666666666667
 25.3333333333333
 25.3333333333333
 25.3333333333333
 1
 24.3333333333333
 5
 5
 5
 5
 52.6666666666667
 48
 40.6666666666667
 10.6666666666667
 9.33333333333333
 .666666666666667
 .666666666666667
 20.3333333333333
 4
 15.6666666666667
 .666666666666667
 1.66666666666667
 .333333333333333
 .333333333333333
 .333333333333333
 .666666666666667
 1
 1
 5.33333333333333
 5.33333333333333
 1.66666666666667
 1.66666666666667
 6.33333333333333
 6
 6
 .333333333333333
 .333333333333333
 1
 1
 .333333333333333
 .666666666666667
 4.66666666666667
 4.66666666666667
 2.33333333333333
 .333333333333333
 .666666666666667
 1.33333333333333
 .666666666666667
 .666666666666667
 .333333333333333
 .333333333333333
 1.33333333333333
 1.33333333333333
 814.666666666667
 43
 41.6666666666667
 41.3333333333333
 41.3333333333333
 .333333333333333
 .333333333333333
 1.33333333333333
 1.33333333333333
 1.33333333333333
 3.66666666666667
 3.66666666666667
 3.66666666666667
 3
 .666666666666667
 .333333333333333
 .333333333333333
 .333333333333333
 .333333333333333
 293.333333333333
 292
 285
 285
 7
 7
 1
 1
 1
 .333333333333333
 .333333333333333
 .333333333333333
 468.333333333333
 468.333333333333
 429.666666666667
 429.666666666667
 34.6666666666667
 34.6666666666667
 .666666666666667
 .666666666666667
 .666666666666667
 .666666666666667
 2.66666666666667
 2.66666666666667
 5
 5
 5
 5
 .666666666666667
 .666666666666667
 .666666666666667
 .666666666666667
 .333333333333333
 .333333333333333
 .333333333333333
 .333333333333333
 12.6666666666667
 1.33333333333333
 1.33333333333333
 1.33333333333333
 .666666666666667
 .666666666666667
 8.66666666666667
 8.66666666666667
 8
 8
 .333333333333333
 .333333333333333
 .333333333333333
 .333333333333333
 2.66666666666667
 2.66666666666667
 2.66666666666667
 2.33333333333333
 .333333333333333
 58
 55
 6.33333333333333
 6.33333333333333
 4.33333333333333
 2
 48.3333333333333
 1.66666666666667
 .666666666666667
 1
 46.6666666666667
 46.6666666666667
 .333333333333333
 .333333333333333
 .333333333333333
 1.66666666666667
 .333333333333333
 .333333333333333
 .333333333333333
 1.33333333333333
 1.33333333333333
 1.33333333333333
 .333333333333333
 .333333333333333
 .333333333333333
 .333333333333333
 1
 1
 1
 1
 240.666666666667
 110
 74.6666666666667
 74.6666666666667
 74.6666666666667
 .333333333333333
 .333333333333333
 .333333333333333
 8.33333333333333
 8.33333333333333
 8.33333333333333
 21
 15.3333333333333
 15.3333333333333
 3.66666666666667
 3.66666666666667
 2
 2
 3.66666666666667
 3.66666666666667
 3.66666666666667
 2
 2
 2
 56.3333333333333
 56.3333333333333
 56.3333333333333
 56.3333333333333
 13
 13
 13
 13
 23
 23
 23
 23
 6
 6
 6
 6
 32.3333333333333
 32.3333333333333
 32.3333333333333
 32.3333333333333
 11.6666666666667
 6.33333333333333
 6.33333333333333
 .333333333333333
 .333333333333333
 5.66666666666667
 3.33333333333333
 .333333333333333
 .333333333333333
 .666666666666667
 1
 .333333333333333
 .333333333333333
 2.66666666666667
 2.66666666666667
 2
 .666666666666667
 .666666666666667
 .666666666666667
 .666666666666667
 .666666666666667
 .666666666666667
 .666666666666667
 .666666666666667
 .666666666666667
 .333333333333333
 .333333333333333
 .333333333333333
 .333333333333333
 1
 1
 .333333333333333
 .333333333333333
 .666666666666667
 .666666666666667
 .666666666666667
 .666666666666667
 .666666666666667
 .666666666666667
 257.666666666667
 257.666666666667
 257.666666666667
 227.333333333333
 227.333333333333
 30.3333333333333
 17.3333333333333
 13
 32.3333333333333
 .333333333333333
 .333333333333333
 .333333333333333
 .333333333333333
 24.6666666666667
 24.6666666666667
 24.6666666666667
 24.6666666666667
 2.66666666666667
 2.66666666666667
 2.66666666666667
 2.66666666666667
 4.66666666666667
 4.66666666666667
 4.66666666666667
 1
 3.66666666666667
 .666666666666667
 .666666666666667
 .666666666666667
 .666666666666667
 .666666666666667
 172
 4.66666666666667
 .333333333333333
 .333333333333333
 .333333333333333
 .333333333333333
 .333333333333333
 .333333333333333
 2.66666666666667
 2.66666666666667
 2.66666666666667
 1.33333333333333
 1.33333333333333
 1.33333333333333
 28.3333333333333
 28.3333333333333
 28.3333333333333
 28.3333333333333
 26.6666666666667
 6
 5.33333333333333
 5.33333333333333
 .666666666666667
 .666666666666667
 17.6666666666667
 15.6666666666667
 15.6666666666667
 2
 2
 3
 3
 3
 56.6666666666667
 31.6666666666667
 31.6666666666667
 31.6666666666667
 25
 25
 25
 16.6666666666667
 16.6666666666667
 16.6666666666667
 16.6666666666667
 30.6666666666667
 21.6666666666667
 21.6666666666667
 21.6666666666667
 9
 7.33333333333333
 7.33333333333333
 1.66666666666667
 1.66666666666667
 .333333333333333
 .333333333333333
 .333333333333333
 .333333333333333
 3.33333333333333
 3.33333333333333
 3.33333333333333
 3.33333333333333
 4.66666666666667
 4.66666666666667
 4.66666666666667
 4.66666666666667
 1.33333333333333
 1.33333333333333
 1.33333333333333
 1.33333333333333
 1.33333333333333
 2.66666666666667
 .333333333333333
 .333333333333333
 .333333333333333
 .333333333333333
 1
 1
 1
 1
 1.33333333333333
 1.33333333333333
 1.33333333333333
 1.33333333333333
 4.33333333333333
 4.33333333333333
 4.33333333333333
 4.33333333333333
 4.33333333333333
 1
 1
 1
 1
 1
 .666666666666667
 .666666666666667
 .666666666666667
 .666666666666667
 .666666666666667
 .666666666666667
 .666666666666667
 .666666666666667
 .666666666666667
 .666666666666667
 .666666666666667
 .666666666666667
 .666666666666667
 .666666666666667
 .666666666666667
 1.33333333333333
 1.33333333333333
 1.33333333333333
 1.33333333333333
 1.33333333333333
 2.33333333333333
 2.33333333333333
 2.33333333333333
 2.33333333333333
 2.33333333333333
 99
 99
 99
 99
 99
 83
 16
