# Supplementary material for: Response of Tomato Rhizosphere Bacteria to Root-Knot Nematodes, Fenamiphos and Sampling Time Shows Differential Effects on Low Level Taxa
Source: Front Microbiol. 2020 Mar 20;11:390. doi: 10.3389/fmicb.2020.00390 (PMC7100632; doi:10.3389/fmicb.2020.00390)
Supplement: FIGURE S2 — Interactive ring-charts (html format) produced with Krona, showing the mean taxonomic repartitions and relative abundance of taxa resulting from the RNAseq analyses, by treatment and sampling times. For treatments codes see legend of Supplementary Figure S1. Files constructed using the mean of three replications, except CON at time T0 (prior to transplants), and FEN-RKN at T2 (6 months), with two replicates each. Unclassified taxa were retained in the analyses. [file Presentation_2.zip › RKN T2 mean.html]

Javascript must be enabled to view this page.

magnitude
 3752.33333333333
 3752.33333333333
 887.333333333333
 7.33333333333333
 7
 7
 7
 .333333333333333
 .333333333333333
 .333333333333333
 467.333333333333
 467.333333333333
 149.333333333333
 149.333333333333
 42.6666666666667
 42.6666666666667
 43.6666666666667
 15.6666666666667
 23.3333333333333
 .333333333333333
 .333333333333333
 .333333333333333
 3.66666666666667
 46.3333333333333
 37
 9.33333333333333
 19.3333333333333
 18
 1
 .333333333333333
 15.6666666666667
 7.66666666666667
 8
 9.66666666666667
 5
 .666666666666667
 4
 .333333333333333
 .333333333333333
 .333333333333333
 .333333333333333
 27
 19.6666666666667
 2.66666666666667
 2
 2.66666666666667
 .333333333333333
 .333333333333333
 12.6666666666667
 .666666666666667
 3.66666666666667
 5.66666666666667
 2.66666666666667
 62.6666666666667
 47.6666666666667
 4.66666666666667
 1.33333333333333
 8.33333333333333
 .666666666666667
 10.6666666666667
 1.33333333333333
 2.66666666666667
 .666666666666667
 6
 2.33333333333333
 2.33333333333333
 4
 4
 5.66666666666667
 5.66666666666667
 1
 .333333333333333
 .666666666666667
 8
 8
 2.66666666666667
 2.66666666666667
 3
 1.33333333333333
 1.66666666666667
 205
 205
 185.666666666667
 185.666666666667
 3
 3
 7.33333333333333
 7.33333333333333
 6.33333333333333
 6.33333333333333
 2.66666666666667
 2.66666666666667
 190
 176.666666666667
 23.6666666666667
 10.6666666666667
 13
 144.666666666667
 144.666666666667
 7
 5.66666666666667
 1.33333333333333
 1.33333333333333
 1.33333333333333
 13.3333333333333
 12.6666666666667
 12.6666666666667
 .333333333333333
 .333333333333333
 .333333333333333
 .333333333333333
 15.6666666666667
 15.6666666666667
 15.6666666666667
 15.3333333333333
 .333333333333333
 2
 .666666666666667
 .666666666666667
 .666666666666667
 1.33333333333333
 1.33333333333333
 1.33333333333333
 1210
 708.333333333333
 163
 108.666666666667
 59.3333333333333
 1
 9
 5
 .333333333333333
 .333333333333333
 .333333333333333
 .333333333333333
 4.66666666666667
 .333333333333333
 .333333333333333
 7
 5.66666666666667
 1
 1
 3
 1.33333333333333
 2
 6.66666666666667
 30
 25.6666666666667
 1
 1.33333333333333
 .666666666666667
 1.33333333333333
 18.3333333333333
 17.6666666666667
 .666666666666667
 5.66666666666667
 5.66666666666667
 .333333333333333
 .333333333333333
 16.6666666666667
 16.6666666666667
 16.6666666666667
 430.666666666667
 430.666666666667
 430.666666666667
 .666666666666667
 .666666666666667
 .666666666666667
 12
 12
 12
 12.6666666666667
 12.6666666666667
 12.6666666666667
 9
 9
 8
 .333333333333333
 .666666666666667
 7.66666666666667
 7.66666666666667
 7.66666666666667
 42
 42
 35
 7
 12.6666666666667
 12.6666666666667
 12.6666666666667
 1.33333333333333
 1.33333333333333
 1.33333333333333
 122.666666666667
 9
 9
 9
 15.3333333333333
 14
 .333333333333333
 .666666666666667
 8.33333333333333
 3
 .333333333333333
 1.33333333333333
 1.33333333333333
 1.33333333333333
 32
 .666666666666667
 .333333333333333
 .333333333333333
 2.66666666666667
 2.33333333333333
 .333333333333333
 2.33333333333333
 1
 1.33333333333333
 4
 2.33333333333333
 1.66666666666667
 7.66666666666667
 7.66666666666667
 11.3333333333333
 11.3333333333333
 1
 1
 2.33333333333333
 2.33333333333333
 4
 2.66666666666667
 2.66666666666667
 1.33333333333333
 1.33333333333333
 49.3333333333333
 45.3333333333333
 45.3333333333333
 1
 .333333333333333
 .666666666666667
 3
 3
 6.66666666666667
 6.66666666666667
 .333333333333333
 .333333333333333
 6
 2.33333333333333
 .333333333333333
 .333333333333333
 2
 .333333333333333
 1.66666666666667
 .666666666666667
 .666666666666667
 .666666666666667
 3.33333333333333
 3.33333333333333
 3.33333333333333
 278
 224.333333333333
 157.666666666667
 122
 3
 1
 3
 6.66666666666667
 9
 1.66666666666667
 11.3333333333333
 66.6666666666667
 22.3333333333333
 44.3333333333333
 15
 15
 14
 1
 1.33333333333333
 1.33333333333333
 1
 .333333333333333
 .333333333333333
 .333333333333333
 .333333333333333
 12.6666666666667
 12.3333333333333
 10.3333333333333
 2
 .333333333333333
 .333333333333333
 17
 .666666666666667
 .666666666666667
 16.3333333333333
 15.3333333333333
 1
 3.33333333333333
 3.33333333333333
 3.33333333333333
 .666666666666667
 .666666666666667
 .666666666666667
 3.33333333333333
 3.33333333333333
 3.33333333333333
 96.6666666666667
 .333333333333333
 .333333333333333
 .333333333333333
 10.6666666666667
 10.6666666666667
 10.6666666666667
 81.3333333333333
 12.3333333333333
 12.3333333333333
 7.66666666666667
 4.33333333333333
 3.33333333333333
 59
 59
 1
 1
 1.33333333333333
 1.33333333333333
 .666666666666667
 .333333333333333
 .333333333333333
 .333333333333333
 .333333333333333
 3.66666666666667
 3.66666666666667
 3.66666666666667
 4.33333333333333
 4.33333333333333
 4.33333333333333
 4.33333333333333
 796.666666666667
 184.666666666667
 184.666666666667
 73.3333333333333
 21.3333333333333
 5.33333333333333
 23.6666666666667
 1.33333333333333
 21.6666666666667
 22.3333333333333
 22.3333333333333
 66.3333333333333
 50.6666666666667
 15.6666666666667
 3.66666666666667
 3.66666666666667
 10
 3.33333333333333
 6.66666666666667
 9
 9
 611.333333333333
 25.6666666666667
 17.6666666666667
 17.6666666666667
 8
 8
 584.666666666667
 549
 547.666666666667
 1.33333333333333
 22
 22
 6.33333333333333
 4.66666666666667
 1.66666666666667
 4.33333333333333
 3.66666666666667
 .666666666666667
 1
 1
 2
 2
 .666666666666667
 .333333333333333
 .333333333333333
 .333333333333333
 .333333333333333
 .333333333333333
 .333333333333333
 .333333333333333
 .666666666666667
 .666666666666667
 .666666666666667
 .666666666666667
 242.666666666667
 1.33333333333333
 1.33333333333333
 1.33333333333333
 1.33333333333333
 231.333333333333
 231.333333333333
 139.333333333333
 139.333333333333
 61.3333333333333
 61.3333333333333
 3.66666666666667
 3.66666666666667
 5
 5
 22
 22
 3.66666666666667
 3.66666666666667
 3.66666666666667
 .666666666666667
 3
 2.66666666666667
 2.66666666666667
 2.66666666666667
 2.66666666666667
 .666666666666667
 .666666666666667
 .666666666666667
 .666666666666667
 3
 3
 2.66666666666667
 2.66666666666667
 .333333333333333
 .333333333333333
 18
 1
 1
 1
 1
 3
 3
 3
 3
 3.66666666666667
 1
 1
 1
 2.66666666666667
 2.66666666666667
 2.66666666666667
 .666666666666667
 .666666666666667
 .666666666666667
 .666666666666667
 .333333333333333
 .333333333333333
 .333333333333333
 .333333333333333
 2.66666666666667
 2.66666666666667
 1
 1
 1.66666666666667
 1.66666666666667
 1.33333333333333
 1.33333333333333
 1.33333333333333
 1.33333333333333
 5.33333333333333
 5.33333333333333
 5.33333333333333
 5.33333333333333
 54.6666666666667
 26
 26
 22
 22
 2.66666666666667
 2.66666666666667
 .333333333333333
 .333333333333333
 .333333333333333
 .333333333333333
 .666666666666667
 .666666666666667
 5
 5
 4.33333333333333
 3
 .333333333333333
 1
 .333333333333333
 .333333333333333
 .333333333333333
 .333333333333333
 20
 20
 18
 3
 .666666666666667
 1
 13.3333333333333
 .666666666666667
 .666666666666667
 1.33333333333333
 1.33333333333333
 1.33333333333333
 1.33333333333333
 .666666666666667
 .333333333333333
 .333333333333333
 .666666666666667
 .666666666666667
 .333333333333333
 .333333333333333
 .333333333333333
 .333333333333333
 2
 2
 2
 2
 431
 199.333333333333
 53
 6
 6
 45.6666666666667
 45.6666666666667
 1.33333333333333
 1.33333333333333
 127.333333333333
 127.333333333333
 127.333333333333
 13.3333333333333
 13.3333333333333
 13.3333333333333
 .666666666666667
 .666666666666667
 .666666666666667
 5
 5
 5
 4.33333333333333
 4.33333333333333
 4.33333333333333
 4.33333333333333
 111
 111
 111
 111
 32.3333333333333
 32.3333333333333
 32.3333333333333
 32.3333333333333
 40.6666666666667
 40.6666666666667
 40.6666666666667
 40.6666666666667
 43.3333333333333
 43.3333333333333
 43.3333333333333
 43.3333333333333
 96.3333333333333
 96.3333333333333
 96.3333333333333
 92.6666666666667
 82.6666666666667
 10
 3.66666666666667
 3.66666666666667
 2.66666666666667
 2.66666666666667
 2.66666666666667
 2.66666666666667
 2.66666666666667
 3
 .333333333333333
 .333333333333333
 .333333333333333
 .333333333333333
 2.66666666666667
 2.66666666666667
 2.66666666666667
 2.66666666666667
 .333333333333333
 .333333333333333
 .333333333333333
 .333333333333333
 .333333333333333
 .333333333333333
 .333333333333333
 .333333333333333
 .333333333333333
 .333333333333333
 4.66666666666667
 4.66666666666667
 4.66666666666667
 4.66666666666667
 4.66666666666667
 2.33333333333333
 2.33333333333333
 2.33333333333333
 2.33333333333333
 2.33333333333333
 2.33333333333333
 2.33333333333333
 2.33333333333333
 2.33333333333333
 2.33333333333333
