# Supplementary material for: Response of Tomato Rhizosphere Bacteria to Root-Knot Nematodes, Fenamiphos and Sampling Time Shows Differential Effects on Low Level Taxa
Source: Front Microbiol. 2020 Mar 20;11:390. doi: 10.3389/fmicb.2020.00390 (PMC7100632; doi:10.3389/fmicb.2020.00390)
Supplement: FIGURE S3 — Venn diagrams showing the number of genera per treatment, at 3 (T1) and 6 months (T2). For control, the number of genera before planting (T0) is also shown. [file Presentation_3.PDF]

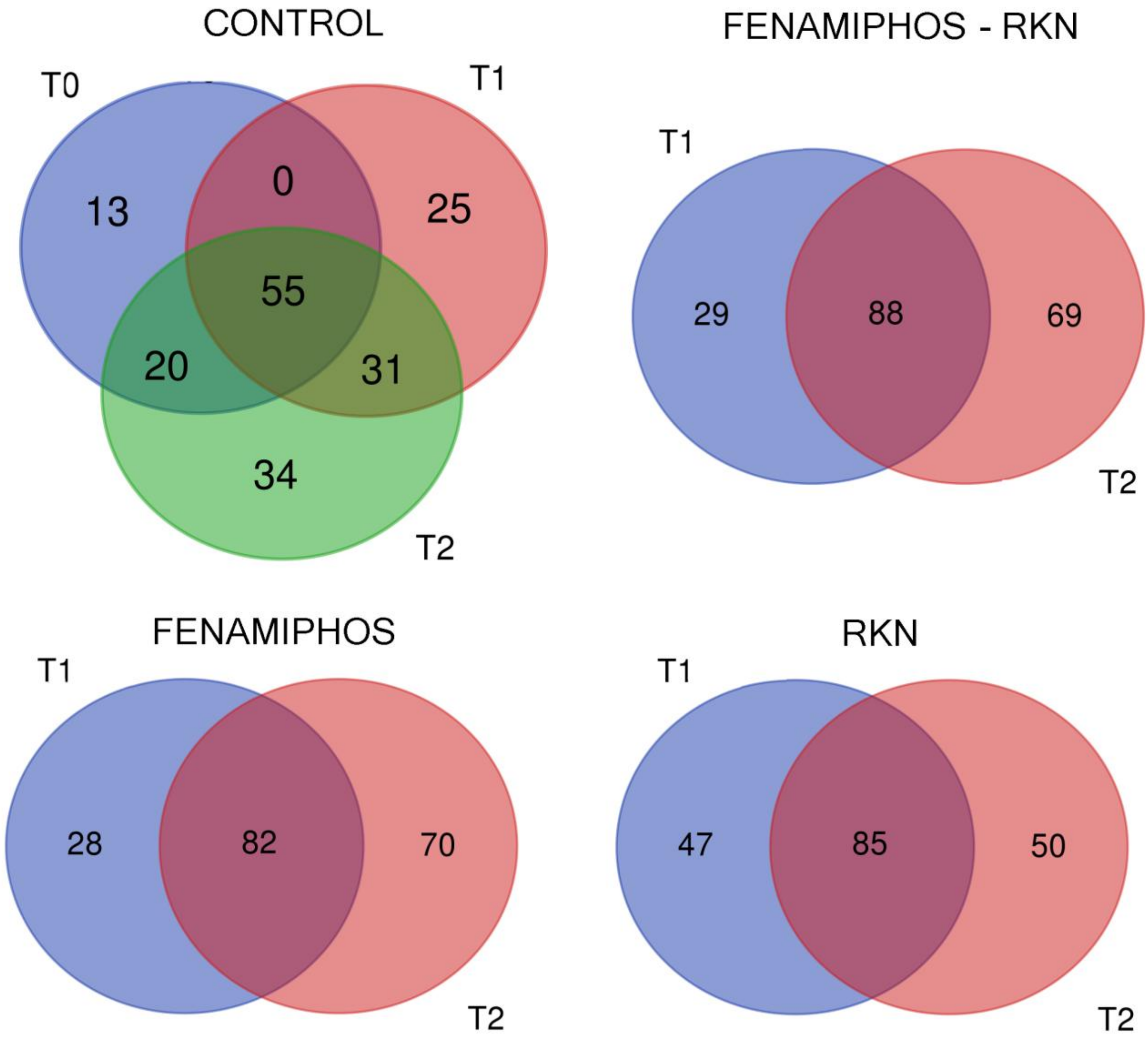

Supplemental Figure S3. Venn diagrams showing the number of genera per treatment and sampling time.
